# Choice of implant combinations in total hip replacement: systematic review and network meta-analysis

**DOI:** 10.1136/bmj.j4651

**Published:** 2017-10-31

**Authors:** José A López-López, Rachel L Humphriss, Andrew D Beswick, Howard H Z Thom, Linda P Hunt, Amanda Burston, Christopher G Fawsitt, William Hollingworth, Julian P T Higgins, Nicky J Welton, Ashley W Blom, Elsa M R Marques

**Affiliations:** 1 Department of Population Health Sciences, Bristol Medical School, University of Bristol, Bristol, UK; 2 Musculoskeletal Research Unit, University of Bristol, Southmead Hospital, Learning and Research Building (Level 1), Bristol BS10 5NB, UK

## Abstract

**Objective** To compare the survival of different implant combinations for primary total hip replacement (THR).

**Design** Systematic review and network meta-analysis.

**Data sources** Medline, Embase, The Cochrane Library, ClinicalTrials.gov, WHO International Clinical Trials Registry Platform, and the EU Clinical Trials Register.

**Review methods** Published randomised controlled trials comparing different implant combinations. Implant combinations were defined by bearing surface materials (metal-on-polyethylene, ceramic-on-polyethylene, ceramic-on-ceramic, or metal-on-metal), head size (large ≥36 mm or small <36 mm), and fixation technique (cemented, uncemented, hybrid, or reverse hybrid). Our reference implant combination was metal-on-polyethylene (not highly cross linked), small head, and cemented. The primary outcome was revision surgery at 0-2 years and 2-10 years after primary THR. The secondary outcome was the Harris hip score reported by clinicians.

**Results** 77 studies were included in the systematic review, and 15 studies (3177 hips) in the network meta-analysis for revision. There was no evidence that the risk of revision surgery was reduced by other implant combinations compared with the reference implant combination. Although estimates are imprecise, metal-on-metal, small head, cemented implants (hazard ratio 4.4, 95% credible interval 1.6 to 16.6) and resurfacing (12.1, 2.1 to 120.3) increase the risk of revision at 0-2 years after primary THR compared with the reference implant combination. Similar results were observed for the 2-10 years period. 31 studies (2888 patients) were included in the analysis of Harris hip score. No implant combination had a better score than the reference implant combination.

**Conclusions** Newer implant combinations were not found to be better than the reference implant combination (metal-on-polyethylene (not highly cross linked), small head, cemented) in terms of risk of revision surgery or Harris hip score. Metal-on-metal, small head, cemented implants and resurfacing increased the risk of revision surgery compared with the reference implant combination. The results were consistent with observational evidence and were replicated in sensitivity analysis but were limited by poor reporting across studies.

**Systematic review registration** PROSPERO CRD42015019435.

## Introduction

Total hip replacement (THR) is one of the most common surgical procedures performed worldwide. In England, Wales, and Northern Ireland, the National Joint Registry recorded that 796 636 THRs were performed between 2003 and 2015.^1^ In the USA an estimated 2.5 million people are living with a hip replacement.^2^ The main indications for elective THR are pain and functional limitations owing to osteoarthritis.^3^

In a primary THR, both the acetabulum and the femoral head are replaced: a metal stem is inserted into the femur with a modular head that articulates with an artificial cup, the acetabular component (metal-on-polyethylene). The most widely used combination of these bearing surfaces comprises a metal femoral head and a polyethylene acetabular cup. It was developed by Sir John Charnley and has been in use since the early 1960s.^4^ Long term survival of these early implants was good, with around 77-81% not needing revision 25 years after primary THR.^5^ An alternative to primary THR is resurfacing hip replacement, in which the acetabulum is replaced with a metal cup, and the femoral head is trimmed and capped with a surface replacement femoral prosthesis. In England and Wales, the use of resurfacings has declined, from 10.8% of hip replacements in 2006 to less than 1% in 2015.^1^ With the increasing use of THR in younger and more active patients where revision rates are higher,^6^ and concerns about the role of polyethylene wear particles in osteolysis and loosening,^5^ new bearing surface materials were introduced.

Current implants have four main combinations of femoral head and acetabular bearing surface materials: metal-on-polyethylene, ceramic-on-polyethylene, ceramic-on-ceramic, or metal-on-metal.^7^ Ceramic-on-metal implants are uncommon. Femoral head sizes vary, typically ranging from 22.225 mm to 50 mm in diameter. The possible fixation techniques (the method of attaching the bearing surface material to the bone) are cemented (when both components are cemented), uncemented (neither component is cemented), hybrid (the femoral stem but not the acetabular cup is cemented), or reverse hybrid (the acetabular cup but not the femoral stem is cemented). In early studies, implant failure after primary THR was attributed to the use of cement, but there is currently little evidence that implant survival rates are superior using other fixation techniques.^8^Figure 1 shows a resurfacing implant; a ceramic-on-ceramic, large head, uncemented implant; and a ceramic-on-polyethylene, small head, cemented implant.

**Figure f1:**
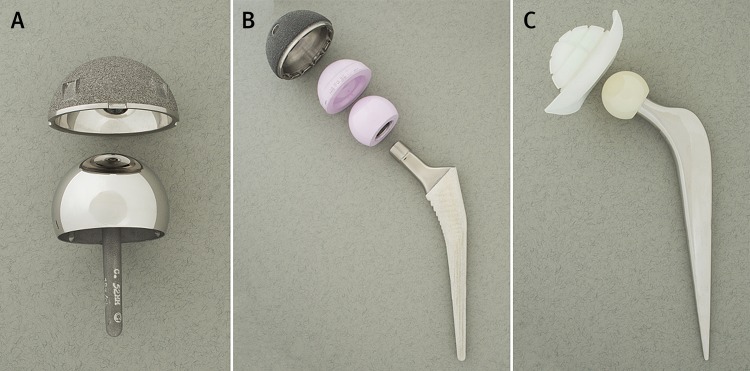
**Fig 1** Illustration of (A) resurfacing implant; (B) ceramic-on-ceramic, large head, uncemented implant (components disassembled); and (C) ceramic-on-polyethylene, small head, cemented implant (head assembled with stem)

When an implant fails (eg, due to loosening, infection, or dislocation) patients may endure severe pain and disability and require surgical revision. Although the key patient expectations of THR are long term reduction in pain, improvement in function, and participation in recreational interests,^5^ these outcomes are inevitably linked to implant survival.^9^ ^10^ Joint registries, with extensive records of patients over time,^11^ play an important role in monitoring the long term performance of implants. An analysis of National Joint Registry data between 2003 and 2011 found higher revision rates for metal-on-metal implants.^12^ In registries, implant combinations are selected for patients based on individual characteristics, making comparisons between implant combinations susceptible to bias. Randomised controlled trials overcome the limitation of patient selection but often lack long term follow-up. Systematic reviews and meta-analyses have been conducted,^13^ ^14^ ^15^ ^16^ but they have not provided a complete overview of randomised evidence on combinations of implant materials, or acquired key information from study authors to allow a focus on revision outcomes.

We present a systematic review of randomised controlled trial evidence comparing rates of revision surgery, and of doctor assessed and patient assessed outcomes of the various bearing surface materials, head sizes, and fixation techniques (implant combinations) used in THR. For the statistical integration of results, we used a network meta-analysis approach, which combines direct and indirect evidence from the implant combinations that are currently available for primary THR. 

## Methods

### Study eligibility and selection

Randomised controlled trials including patients aged 18 years or older with a diagnosis of osteoarthritis in the majority (>50%) of patients, comparing different bearing surface materials (including resurfacing) or head sizes in primary total hip replacement (THR) were eligible. Web appendix 1 shows the possible implant combinations. We categorised head sizes as large (≥36 mm in diameter) or small (<36 mm). Polyethylene materials were further classified as highly cross linked (a newer generation of polyethylene) or not highly cross linked (including conventional polyethylene and other types of polyethylene). We excluded studies of patients receiving simultaneous bilateral THR, emergency surgery, or revision hip replacement.

The primary outcome was first revision surgery after primary THR or resurfacing. The secondary outcomes were the Harris hip score reported by clinicians,^17^ and the Oxford hip score^18^ and Western Ontario and McMaster Universities osteoarthritis index score (WOMAC)^19^ based on patient report.

We undertook a systematic search of the literature in February 2015 (updated in July 2016). Web appendix 2 shows the search strategy as applied in Medline. This was tailored to each database, and used in Medline, Embase, and The Cochrane Library. We also searched the clinical trials databases ClinicalTrials.gov, WHO International Clinical Trials Registry Platform, and the EU Clinical Trials Register. We checked reference lists of identified studies and tracked citations of key articles in Web of Science. Websites of orthopaedic conferences since January 2012 were examined to identify current unpublished studies. Our searches had no language restrictions.

We registered our systematic review prospectively in PROSPERO (CRD42015019435). We formulated the research question according to the PICO (population, intervention, comparison, and outcome) principle.^20^ The systematic review methods were based on those recommended by Cochrane,^21^ and reporting was in accordance with the Reporting Items for Systematic Reviews and Meta-Analyses (PRISMA) guidelines.^22^ Further details of the methods can be found in the published protocol.^7^

### Data collection and assessment of risk of bias

Two reviewers independently screened titles and abstracts and extracted data from the included studies. They contacted authors of published studies, protocols, and trial register entries for additional information when necessary. The reviewers independently assessed the risk of bias using the Cochrane risk of bias tool.^21^ They noted key explanations for a high risk of bias assessment.

### Data synthesis and statistical analysis

To define interventions we considered combinations of bearing surface materials, head sizes, and fixation techniques. We excluded studies using bearing surface materials that are not commonly used in clinical practice, and studies assessed at high risk of bias for the outcomes of interest.

Using the *network *suite of commands for Stata,^23^ we generated network plots for each outcome to illustrate which interventions had been compared directly in the included studies. Network meta-analysis is an extension of standard meta-analysis to compare multiple treatments based on randomised controlled trial evidence, which forms a connected network of comparisons.^24^ Treatment effect estimates from network meta-analysis exploit both the direct comparisons within trials and the indirect comparisons across trials. As the reference implant, we chose a widely used combination, the metal-on-polyethylene (not highly cross linked), small head, cemented implant.^4^ ^6^ All network meta-analyses were implemented in a bayesian framework using OpenBUGS software (version 3.2.3).^25^ We used fixed effect models, as few replications of each implant comparison were available for analyses, and assessed consistency between direct and indirect evidence by comparing the fit of the consistency model with the fit of an inconsistency model.

The effect measure for revision surgery events was the hazard ratio. We modelled the data using a binomial likelihood and a logistic link function. The unit of analysis was the hip. Studies reported at different time points, with several studies reporting at multiple times (see web appendix 3), all of which were included in the analyses. Based on clinical expertise and the literature, we considered two periods after primary THR for implant failure: 0-2 years and 2-10 years. Ten years was often the longest follow-up available in the literature and an acceptable survival duration for clinical advisors of the study. We assumed piecewise constant hazard ratios over the two periods but assumed the log hazard ratios in the later period were related to those in the earlier period by a random walk model.^26^ This model assumes that log hazard ratios in the second and third period are normally distributed around those in the first and second periods, respectively. Web appendix 3 provides details.

In sensitivity analyses, we considered a different cut-off point for the change in the hazard ratio (0-5 and 5-10 years). Revision surgery is a rare outcome and large numbers of primary surgeries or a long follow-up period after surgery is often required for events to occur. To achieve stable results, we implemented continuity corrections by adding 0.5 revision events to both arms of studies with zero revisions in some (but not all) arms, and if stable results were still not obtained, we excluded studies with zero counts.

The secondary outcomes of Harris hip score reported by the clinician and other outcomes reported by the patient were continuous variables. The unit of analysis for these outcomes was the patient. We computed differences in mean score between treatment groups at the longest follow-up time point, using a normal likelihood and an identity link function.

Owing to limitations in data availability, statistical integration of results for other outcome measures—namely, those reported by the patient, as well as the adjustment for age in meta-regression, was not possible.

### Patient involvement

Patients and hip surgeons were actively involved in designing the study and interpreting and disseminating the results. AB runs two patient involvement groups at the Musculoskeletal Research Unit, Southmead Hospital: one group consisting of nine people with musculoskeletal conditions, most of whom have had joint replacements, including THR; and a second consisting of four patients who have had THR revision surgery. Both groups provided continuous feedback to the team throughout the study. Two lay members were invited to two steering group meetings and provided patient views on ongoing works. EMRM and AB also met at the start and end of the project with hip surgeons at Southmead Hospital for additional clinical advice on study design and impact of findings for clinical practice. Whether the studies included in the review had any patient involvement was not evaluated.

## Results

### Included studies

We identified 3066 articles through database searches, 1322 from searching clinical trial registries, and 18 from reference lists, giving a total of 4406 articles (fig 2). After an initial screen of titles and abstracts where we excluded studies clearly not relevant, 218 articles were considered potentially relevant to the review and full papers were obtained. Two reviewers independently assessed full papers, and 77 unique randomised controlled trials described in 136 articles were included in the review. Figure 2 summarises the reasons for study exclusions. Web appendix 4 summarises the characteristics and references of studies.

**Figure f2:**
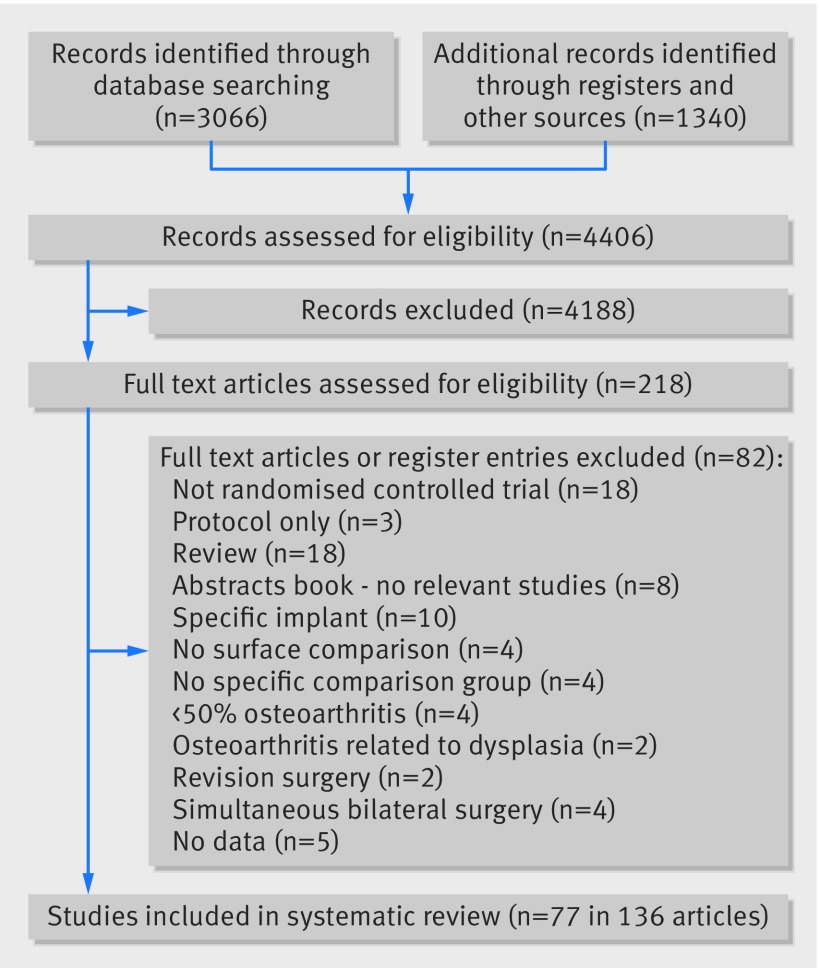
**Fig 2** Systematic review flow diagram

The 77 randomised controlled trials included patients from 83 centres, geographically dispersed, mainly throughout North America and European countries. Studies included between two and five arms (169 arms in total). Allocation to randomised implant combinations ranged from 5 to 349 hips (mean 68), with a total of 11 700 hips randomised. We only included data from time points at which follow-up was attempted for all patients in the trial. The mean age of patients in randomised groups ranged from 47.1 to 72.6 years (median 61 years) and the percentage of female patients ranged from 10.8% to 100% (median 58% females). Studies in the review were powered on a range of different outcomes, most commonly hip scores reported by clinicians and patients. No studies were powered to detect differences in revision rates.

Web appendix 5 summarises our risk of bias assessments, including justifications for considering studies to be at high risk of bias. The assessments were undertaken from the perspective of the primary outcome (revision surgery), although they also apply to the secondary outcome (Harris hip score reported by clinicians), as both outcomes involve clinical judgment and are measured at similar time points across studies. Our assessment suggested that risk of bias was low in 30 studies, high in 12, and unclear in 35. Risk of bias related to high losses to follow-up, differences in follow-up between randomised groups, baseline differences in groups, and selective reporting. Unclear risk of bias related to limited reporting of methods and blinding. Surgeons are often not blinded to the allocation of interventions in surgical trials. Nonetheless, revision is an objective outcome, and hence we decided to keep studies at unclear risk of bias in the analyses.

Out of the 77 studies included in the review, three did not consider commonly used implant combinations (two were rarely used ceramic-on-metal implants and one used the discontinued Hylamer polyethylene), and 14 did not provide enough information to define an implant combination (eg, comparison of head sizes both classified as small in our study), leaving 60 studies available for statistical integration of results. The maximum follow-up periods in these 60 studies ranged between three months and 13 years (median 2 years).

### Primary outcome: revision surgery

Thirty of the 60 studies provided pairwise comparisons of different implant combinations, after excluding 17 studies that did not report revision surgeries and 13 studies with zero events in all arms. Web appendix 6 shows the results for the pairwise comparisons across those 30 studies (supplementary figs 1 and 2). There is no strong evidence of a difference in rates of revision surgery at 0-2 years after primary total hip replacement (THR). Most effect estimates had wide confidence intervals, and all of them included the null value (hazard ratio of 1). More studies are available for the 2-10 years period; still there is no clear evidence, in the studies with a low risk of bias, for superiority of one implant combination over another.

Out of the 30 studies providing pairwise comparisons, 15 (3177 randomised hips) provided data for network meta-analysis of revision surgery after primary THR. Exclusions consisted of five studies assessed to be at high risk of bias for the primary outcome, five studies of implant combinations disconnected from the main network, and five studies with zero events in at least one arm (no revisions reported). These studies had small samples or short term follow-up, or both, causing convergence problems.

Figure 3 shows the network plots at 0-2 years and 2-10 years after primary THR, with the reference implant combination at the bottom of the plots. These illustrate the implant combination comparisons that were made within the 15 included studies. Thick lines represent comparisons that were reported in two studies; the number of hips contributing to each comparison is also displayed. Only two of these studies contributed data to both periods. Only small head implant combinations were available for 0-2 years. The random walk model allowed us to analyse all studies simultaneously for all implant combinations for both periods.

**Figure f3:**
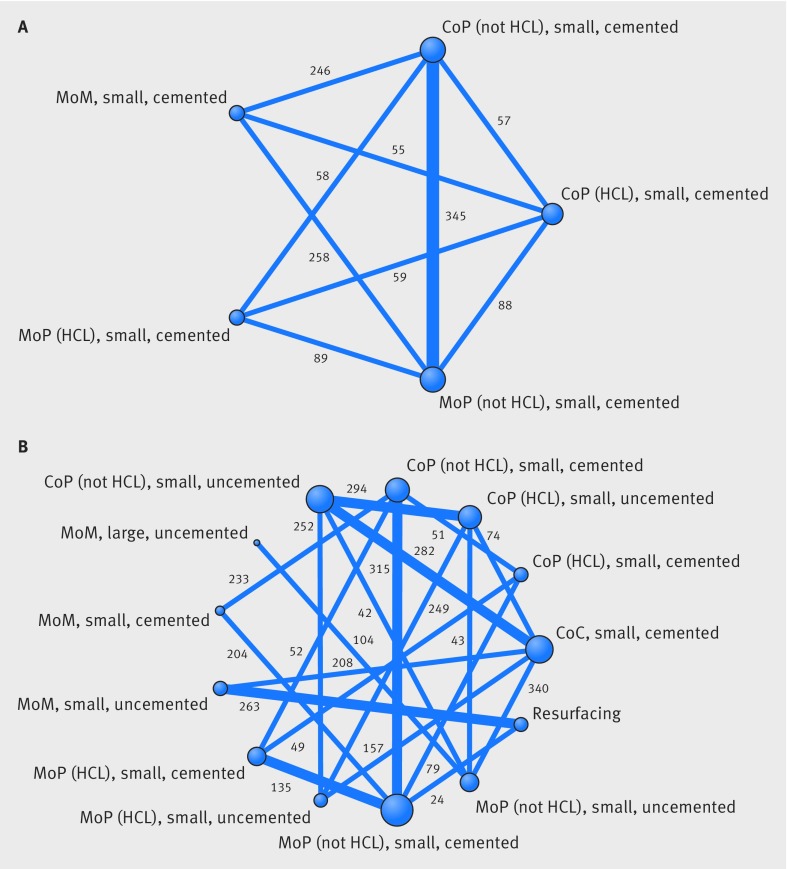
**Fig 3** Network plots for revision at (A) 0-2 years and (B) 2-10 years after primary total hip replacement. The presence of a line between implant combination nodes indicates that the implant combinations had been compared directly within a trial. Node size and line thickness are proportional to the number of studies contributing to each intervention and comparison, respectively. The number of hips contributing to each comparison is displayed. CoP=ceramic-on-polyethylene; HCL=highly cross linked; MoP=metal-on-polyethylene; MoM=metal-on-metal; CoC=ceramic-on-ceramic

Figure 4 shows the results of the network meta-analysis for revision surgery at 0-2 years and 2-10 years after primary THR. Metal-on-metal, small head, cemented implants and resurfacing hip replacements increased the risk of revision surgery compared with the reference implant combination (metal-on-polyethylene (not highly cross linked), small head, cemented) for both periods. Although estimates are imprecise, we found that the metal-on-metal, small head, cemented implant (hazard ratio 4.4, 95% credible interval 1.6 to 16.6) and resurfacing (12.1, 2.1 to 120.3) increase the risk of revision surgery compared with the reference implant combination at 0-2 years after primary THR. We observed similar results for the 2-10 years period. All implant combinations yielded, on average, higher rates of revision surgery than the reference implant combination, except for ceramic-on-ceramic, small head, uncemented implants, but the 95% credible intervals for both periods were wide and included the null value (hazard ratio of 1).

**Figure f4:**
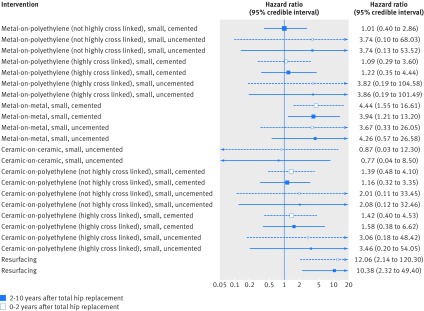
**Fig 4** Network meta-analyses for random walk model for revision at 0-2 years and 2-10 years after primary total hip replacement. Hazard ratios greater than 1 favour the reference implant combination (metal-on-polyethylene (not highly cross linked), small, cemented)

Analysing each period separately (without the random walk model, but using a fixed effects model) and considering a different cut-off point for the period after primary THR (0-5 and 5-10 years) yielded similar results (see supplementary figs 3-5 in web appendix 6). There was no evidence of inconsistency between direct and indirect evidence based on both comparisons of consistency and inconsistency model fits and comparison of pairwise (supplementary figs 1 and 2 in web appendix 6) and network meta-analysis (fig 4) results, although this may be due to the high levels of imprecision. In a separate sensitivity analysis, we excluded resurfacing hip replacements and arms including fewer than 20 hips. Both approaches had an impact on revision surgery at 2-10 years, resulting in a disconnected network that excluded some relevant implant combinations (such as metal-on-metal, small head, uncemented) and showed no differences among the implant combinations that remained in the main network.

### Secondary outcome: Harris hip score reported by clinicians

In 31 of the 62 studies (2888 patients) there was enough information to compare the Harris hip score for different implant combinations, with follow-up periods between 3 and 101 months **(**median 24.5 months). Figure 5 shows the network plot of 19 connected implant combinations and figure 6 presents the network meta-analysis results for the secondary outcome.

**Figure f5:**
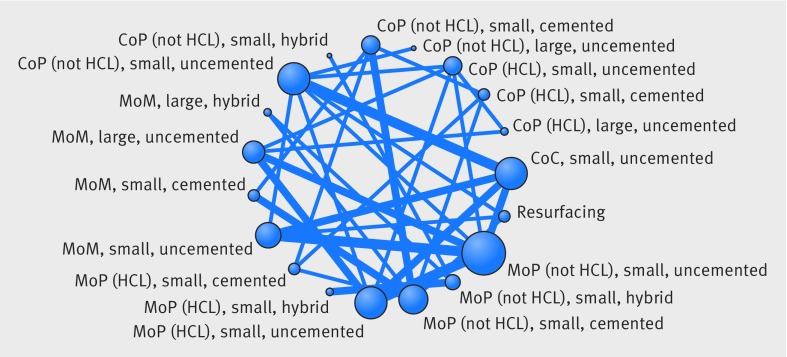
**Fig 5** Network plot for Harris hip score. Presence of a line between implant combination nodes indicates that the implant combinations had been compared directly within a trial. Node size and line thickness are proportional to the number of studies contributing to each intervention and comparison, respectively. CoP=ceramic-on-polyethylene; HCL=highly cross linked; CoC=ceramic-on-ceramic; MoP=metal-on-polyethylene; MoM=metal-on-metal

**Figure f6:**
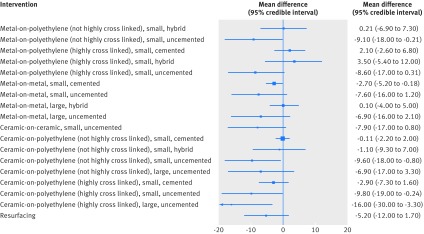
**Fig 6** Network meta-analysis results for Harris hip score. Mean differences less than zero favour the reference implant combination (metal-on-polyethylene (not highly cross linked), small, cemented)

There is no evidence that any implant combination provides a better outcome on the Harris hip score than the reference implant combination. Our estimates provide some evidence that the reference implant combination is associated with a better Harris hip score than metal-on-metal, small head implants, and most uncemented implants (fig 6). As the distribution of Harris hip scores was negatively skewed, we ran additional analyses using a log-normal distribution, but this did not impact the results.

### Outcomes reported by patients

Twenty eight studies reported disease or joint specific outcomes reported by patients (Western Ontario and McMaster Universities osteoarthritis index (WOMAC) and Oxford hip score). A small number of studies reported other generic patient outcome measures. Data on WOMAC and Oxford hip score outcomes was of poor quality and inconsistently reported across studies (different scoring methods; missing measures for average, variance, or sample size; report of a subset of the scale; and a wide range of follow-up points). We were therefore unable to proceed with the statistical integration of these results. After excluding four studies at high risk of bias, we summarised WOMAC and Oxford hip score outcomes narratively (see web appendix 7). In multiple randomised controlled trials there was no suggestion that outcomes reported by patients differed between metal-on-polyethylene and other combinations of bearing surface material (ceramic-on-ceramic, metal-on-metal, or ceramic-on-polyethylene) or between metal femoral heads in combination with different types of polyethylene acetabular surfaces. Similarly, outcomes reported by patients did not differ when a ceramic implant was used in combination with different types of polyethylene acetabular surface, or with a ceramic surface. One study^27^ reported a better outcome by patient self report in the long term for those receiving hip resurfacing compared with metal-on-metal implants. This was not confirmed in two other randomised controlled trials.^28^ ^29^ Other combinations were explored in single randomised controlled trials, with no suggestion that any specific bearing surface material combination gave a more favourable outcome reported by patients in the long term.

## Discussion

In this comprehensive systematic review and network meta-analysis of a wide range of implant combinations using randomised evidence, we found no evidence that newer implant combinations are superior to the reference implant combination (metal-on-polyethylene (not highly cross linked), small head, cemented) both for risk of revision surgery and for the Harris hip score reported by clinicians. Resurfacing and metal-on-metal, small head, cemented implants increase the risk of revision surgery compared with the reference implant combination. The studies included in our network meta-analysis pre-date the recall of metal-on-metal implants, as patients were followed up until 2010 at the latest. Our findings were similar across the 0-2 years and 2-10 years after primary total hip replacement (THR) periods and were replicated in a variety of sensitivity analyses.

A previous systematic review of randomised controlled trials also found that resurfacings were more likely to be revised than metal-on-polyethylene implants.^13^ This evidence is also supported by observational studies using the National Joint Registry cohort, which found that resurfacings and metal-on-metal implant combinations increased the risk of revision surgery compared with metal-on-polyethylene combinations.^12^ ^30^ These findings led to a change in the UK National Institute for Health and Care Excellence guidance on the use of prostheses with a risk of revision surgery at 10 years after primary THR from below 10% to below 5%.^31^ ^32^ Registry data also suggests that ceramic-on-ceramic, large head implants might improve implant survival compared with metal-on-polyethylene implants, but this finding is prone to selection bias, and data from randomised controlled trials were not available in our study to test this hypothesis. One observational study found lower risks of revision surgery for implant combinations with highly cross linked polyethylenes,^33^ whereas we found no evidence in favour of highly cross linked polyethylenes compared with other polyethylenes. Our finding may result from a lack of data in our network meta-analysis to compare outcomes, but is in line with published evidence from six national and regional registries.^34^

### Strengths and weaknesses of this study

We performed an extensive literature search on combinations of hip prosthetic implant combinations, which include all available randomised controlled trial evidence, irrespective of language, sample size, and duration of follow-up. Revision surgery after primary THR is a rare outcome. For statistical integration of results, we had to exclude some smaller studies without revision events to obtain stable results. Few studies directly compared the same implant combinations, hence we could not estimate and assess between study heterogeneity using random effects models. Furthermore, we were unable to explore the impact of potential effect modifiers, such as age, sex, and other patient characteristics, and differences in surgical treatment, perioperative care, and rehabilitation between studies using meta-regression. Most studies focused on other clinical outcomes than revision surgery. They were not powered to detect differences in rates of revision surgery, with revision surgery often reported as a minor, even incidental, outcome. Other studies considered early failures as surgical failures and not implant failures, and excluded patients from further follow-up. Similar limitations have been noted previously.^13^

Our study highlights the shortcomings of the current evidence. Improving the quality of scientific reporting remains the main goal of the Core Outcome Measures in Effectiveness Trials (COMET)^35^ and similar initiatives across many specialties, including hip surgery,^36^ to increase the impact of primary study reports and systematic reviews and to inform good clinical practice. Although we were unable to analyse outcomes reported by the patients in our study, a moderate to high correlation between outcomes reported by clinicians and patients has been described previously.^37^ Joint pain and joint function reported by patients are now recognised as key outcomes of hip and knee replacement and have been widely collected in recent studies. Future studies should report core outcomes similar to those described in the Initiative on Methods, Measurement, and Pain Assessment in Clinical Trials (IMMPACT), which recommends reporting of pain, physical functioning, emotional functioning; participants’ ratings of global improvement, satisfaction with treatment, and disposition; and serious adverse events.^38^

### Conclusion

Identifying the most appropriate implant combination for use in THR is a recognisable priority^39^ given the number of patients undergoing primary THR worldwide. Selection of implant combination type is determined by multiple factors: surgeon preference, such as surgical training and skills; patient factors, such as age and bone shape; healthcare provider factors, such as cost pressures on hospitals and availability of implant combinations; and ultimately the available evidence in the literature. Despite the large number of THRs performed annually worldwide, the number of implant combinations available on the market, and the number of studies performed by manufacturers comparing hip implants, there is still little evidence available to allow integration of results and inform decision making. Consequently, clinical practice and guidance relies on registry evidence, which is more prone to bias. This review highlights the need for new randomised controlled trials with rigorous reporting on core, adequately powered outcomes (possibly within an Idea, Development, Exploration, Assessment, Long-term study (IDEAL) collaboration^40^), to inform decision making. Our future work includes liaising with international collaborations for new multiarm randomised controlled trial comparisons of implant combinations. Our findings have implications for clinical practice, reassuring clinicians and patients that there is no evidence that newer implant combinations may be superior to metal-on-polyethylene implants, irrespective of head size and fixation technique.

**What is already known on this topic**

Observational evidence using joint registry data suggests that resurfacing hip replacements and metal-on-metal implant combinations fail at a higher rate than metal-on-polyethylene, small head, cemented implants
Joint registry data also suggest that newer ceramic-on-ceramic, large head implant combinations might improve implant survival compared with standard implant combinations

**What this study adds**

A synthesis of randomised evidence suggests that resurfacings and metal-on-metal, small head, cemented implants increase the risk of revision surgery compared with metal-on-polyethylene, small head, cemented implants
There was no evidence that newer implant combinations, such as ceramic-on-ceramic implants are superior to the metal-on-polyethylene, small head, cemented implants

## Web Extra

Extra material supplied by the author

## Notes

This article summarises findings from the Hip Implant Prosthesis Study (HIPS) study. We thank the HIPS steering group members: Keith Abrams (University of Leicester), Mark Wilkinson (University of Sheffield and National Joint Registry scientific committee), Mark Pennington (Kings College London), David Peacock (lay member), and Michael Nicholson (lay member) for methodological advice, and the Patient Experience Partnership in Research group (PEP-R) for patient views. We also thank Alison Richards for help in the development of the search strategy and Katie Warner for administrative support. Copyright: This work is subject to Crown Copyright.

Contributors: EMRM, ADB, AWB, NJW, and WH conceived the project. RLH and ADB selected articles for inclusion, extracted data, and assessed risk of bias. RLH, ADB, JALL, EMRM, AWB, JPTH, WH, and NJW selected studies for inclusion. HHZT and JALL planned the statistical analyses with advice from NJW, JPTH, WH, and EMRM. JALL performed the network meta-analysis with advice from HHZT and CGF. AWB and LPH provided clinical expertise throughout, and AB and EMRM liaised with patient and surgeons for their involvement. EMRM, JALL, ADB, RLH, and AWB wrote the first draft of the paper and all authors revised it critically for important intellectual content. All authors have approved this version for publication. EMRM is the guarantor.

Ethical approval: Not required.

Data sharing: No additional data available.

Transparency: The lead authors (EMRM, ADB, and JALL), affirm that the manuscript is an honest, accurate, and transparent account of the study being reported; that no important aspects of the study have been omitted; and that any discrepancies from the study as planned and registered have been explained.
