# Supplementary material for: Choice of implant combinations in total hip replacement: systematic review and network meta-analysis
Source: BMJ. 2017 Oct 31;359:j4651. doi: 10.1136/bmj.j4651 (PMC5683044; doi:10.1136/bmj.j4651)
Supplement: Supplementary file 1 — Appendix: Supplementary materials [file lopj039521.ww1.pdf]

Appendix 1: Possible combinations of bearing surfaces, head sizes and fixation

Femoral head-on-acetabular surface bearing combination	Head size	Fixation
Ceramic-on-ceramic (CoC)	Large ≥36mm	With cement
	Small <36mm	Without cement
		Hybrid
		Reverse hybrid
Metal-on-metal (MoM)	Large ≥36mm	With cement
	Small <36mm	Without cement
		Hybrid
		Reverse hybrid
Metal-on-polyethylene (MoP, not HCL)	Large ≥36mm	With cement
	Small <36mm	Without cement
		Hybrid
		Reverse hybrid
Metal-on-polyethylene (MoP, HCL)	Large ≥36mm	With cement
	Small <36mm	Without cement
		Hybrid
		Reverse hybrid
Ceramic-on-polyethylene (CoP, not HCL)	Large ≥36mm	With cement
	Small <36mm	Without cement
		Hybrid
		Reverse hybrid
Ceramic-on-polyethylene (CoP, HCL)	Large ≥36mm	With cement
	Small <36mm	Without cement
		Hybrid
		Reverse hybrid
Metal-on-metal resurfacing	Large	

HCL: highly cross-linked
