# Supplementary material for: Choice of implant combinations in total hip replacement: systematic review and network meta-analysis
Source: BMJ. 2017 Oct 31;359:j4651. doi: 10.1136/bmj.j4651 (PMC5683044; doi:10.1136/bmj.j4651)
Supplement: Supplementary file 2 — Appendix: Supplementary materials [file lopj039521.ww2.pdf]

Appendix 2: Medline search strategy

1. hip prosthesis.mp. or Hip Prosthesis/
2. Arthroplasty, Replacement, Hip/ or Hip Prosthesis/ or total hip.mp.
3. Arthroplasty, Replacement, Hip/ or Hip Prosthesis/ or hip arthroplasty.mp.
4. Hip Prosthesis/ or Arthroplasty, Replacement, Hip/ or hip replacement.mp.
5. (THR or THA).tw.
6. Hip Joint/
7. Or/1-6

8. randomized controlled trial.pt. or randomized controlled trial/ or Randomized Controlled Trials as Topic/
9. controlled clinical trial.pt.
10. randomi#ed.ab.
11. randomly.ab,ti
12. random.ti
13. random*.tw
14. trial.ab,ti
15. clinical trials as topic.sh
16. MEDLINE.tw.
17. Systematic review.tw.
18. Meta analysis.pt.
19. Or/8-18

20. ceramic.mp. or Ceramics/
21. Zirconium.mp. or Zirconium/
22. alumina.mp. or Aluminum Oxide/
23. oxinium.tw
24. cerasul.tw.
25. CoM.tw.
26. CoC.tw.
27. CoP.tw.
28. polyethylene.mp. or Polyethylene/

29. plastic.mp. or Plastics/
30. UHMWPE.mp.
31. XLPE.mp.
32. X3.mp.
33. metal.mp.
34. MoM.mp.
35. cobalt.mp. or Cobalt/
36. Chromium Alloys/ or Chromium/ or chrome.mp.
37. metallic.mp.
38. metasul.tw.
39. MoP.mp.
40. hard on hard.mp.
41. Hard-on-soft.mp.
42. alloy.mp. or Alloys/
43. bearing.mp
44. (resurf$ or re-surf$).mp. [mp=ti, ab, rw, sh]
45. 22 mm.tw or 22mm.tw
46. 24 mm.tw or 24mm.tw
47. 26 mm.tw or 26mm.tw
48. 28 mm.tw or 28mm.tw
49. 30 mm.tw or 30mm.tw
50. 32 mm.tw or 32mm.tw
51. 34 mm.tw or 34mm.tw
52. 36 mm.tw or 36mm.tw
53. 38 mm.tw or 38mm.tw
54. 40 mm.tw or 40mm.tw
55. 42 mm.tw or 42mm.tw
56. 44 mm.tw or 44mm.tw
57. 46 mm.tw or 46mm.tw
58. 48 mm.tw or 48mm.tw
59. 50 mm.tw or 50mm.tw
60. 52 mm.tw or 52mm.tw

61. 54 mm.tw or 54mm.tw
62. 56 mm.tw or 56mm.tw
63. 58 mm.tw or 58mm.tw
64. 60 mm.tw or 60mm.tw
65. (BHR or Conserve Plus or Durom or Cormet or ASR or ReCap).ti,ab
66. head size.mp.
67. femoral adj2 size
68. large adj2 head
69. femoral adj2 head
70. or/20-69
71. 7 and 19 and 70
