# Supplementary material for: Choice of implant combinations in total hip replacement: systematic review and network meta-analysis
Source: BMJ. 2017 Oct 31;359:j4651. doi: 10.1136/bmj.j4651 (PMC5683044; doi:10.1136/bmj.j4651)
Supplement: Supplementary file 3 — Appendix: Supplementary materials [file lopj039521.ww3.pdf]

Appendix 3: Additional details of the random walk model

A. Data structure

Study	No. Arms	Time points (in months)
Bjorgul (2013)	3	24, 60, 84
D'Antonio (2002)	2	33
Digas (2003)	2	24, 36, 60, 120
Howie (2005)	2	60, 100
Morison (2014)	4	60
Hamilton (2010)	2	30
Kadar (2011)	4	12, 24, 60
Ochs (2007)	2	3,101
Pabinger (2003)	2	7, 24
Bal (2005)	2	24
Bascarevic (2010)	2	50
Beaupre (2013)	2	12, 60, 120
Desmarchelier (2013)	2	60, 98
Jassim (2015)	3	60
Girard (2006)	2	54, 93

B. Modelling details

We used a binomial likelihood and a logistic link function to model the number of revisions in the jth observed time period of the kth arm or the ith study:

$$r_{ikj} \sim Binomial(p_{ikj}, n_{ikj})$$

p_{ikj} is the probability of revision; and n_{ikj} is the number of hips at risk at the start of observation period j.

The probability of revision p_{ikj} is defined by the exposure of the hips in each of the two time periods:

$$p_{ikj} = 1 - e^{-\theta_{ikj}}$$

As the time period for observation j may not match the time periods of the model (0-2 and 2-10 years), we split the contribution to the total hazard between the model time periods:

$$\theta_{ikj} = \lambda_{ik}^{0-2} E_{ikj}^{0-2} + \lambda_{ik}^{2-10} E_{ikj}^{2-10},$$

where E_{ikj}^{0-2} is exposure of observation j in 0-2 years; E_{ikj}^{2-10} is exposure of observation j in 2-10 years; and λ_{ik}^{0-2} and λ_{ik}^{2-10} are the hazards in these two time periods.

Log hazard ratios (treatment effects) in $x = 0 - 2$ and $x = 2 - 10$ years in study i and arm k were modelled as

$$\log(\lambda_{ik}^{x}) = \mu_{it} + d_{tik}^{x}.$$

The random walk model assumes treatment effects for implant t in period 2-10 were assumed normally distributed around effects in 0-2 years with between time period variance σ_d shared across implants:

$$d_t^{2-10} \sim N(d_t^{0-2}, \sigma_d)$$

More complex structural assumptions would not be identifiable as there are only two time periods. Vague prior distributions were assumed for treatment effects in period 0-2 years:

$$d_t^{0-2} \sim N(0, \sigma = \sqrt{1000})$$

We also ran several sensitivity analyses where we modelled the treatment effects as

$$\log(\lambda_{ik}^x) = \mu_{it} + d_{t_{ik}}^{0-2} + \varphi_{t_k}^x$$

where $\varphi_{t_k}^{0-2} = 0$, and we explored three different parameterisations for $\varphi_{t_k}^{2-10}$, namely

$\varphi_{t_k}^{2-10} = 0$, $\varphi_{t_k}^{2-10} = f$ and $\varphi_{t_k}^{2-10} \sim N(f, \sigma_\varphi)$, with vague priors $f \sim N(0, \sqrt{1000})$ and $\sigma_\varphi \sim U(0{,}5)$. The results showed the same trends but with greater precision, although the heterogeneity increased and DIC statistics supported our base case model.
