# Supplementary material for: Choice of implant combinations in total hip replacement: systematic review and network meta-analysis
Source: BMJ. 2017 Oct 31;359:j4651. doi: 10.1136/bmj.j4651 (PMC5683044; doi:10.1136/bmj.j4651)
Supplement: Supplementary file 4 — Appendix: Supplementary materials [file lopj039521.ww4.pdf]

Appendix 4: Characteristics of included studies

Study<br>Key paper (all study references)<br>Date<br>Country	Group<br>Bearing combination<br>Head size<br>Fixation	Number of hips randomised<br>% bilateral (if reported)	Mean age (SD or range)<br>% female<br>% osteoarthritis (OA)<br>Mean BMI (SD)	Outcome measures relevant to review<br>Follow up times (months)
Amanatullah et al. 2011* (1, 2)<br>1999 – 2001<br>USA	1. CoC<br>Alumina: Alumina<br>28-32mm<br>Uncemented	196<br>15.3% bilateral	50.4 years (12.8)<br>36.1% female<br>88.8% OA<br>BMI 29.6 (12.4)	Revision, HHS, SF-12<br>3, 6, 12, 24, 48, 60 months
	2. CoP<br>Alumina: UHMWPE<br>28mm<br>Uncemented	161<br>9.3% bilateral	54.7 years (12.9)<br>42.5% female<br>87.6% OA<br>BMI 28 (5.1)	
Ando et al. 2015 (3)<br>2009-2011<br>Japan	1. MoM<br>Co-Cr-Mo: Co-Cr-Mo<br>28-32mm<br>Uncemented	91<br>3.3% bilateral	66.2 years (9.0)<br>74.7% female<br>92.3% OA<br>BMI 23.1 (3.6)	HHS, EQ-5D, UCLA activity score<br>3, 6, 12, 24 months
	2.MoM<br>Co-Cr-Mo: Co-Cr-Mo<br>38-52mm<br>Uncemented	98<br>1.02% bilateral	65.4 years (8.6)<br>77.6% female<br>82.6% OA<br>BMI 23.9 (3.7)	
Ayers et al. 2009 (4)<br>Not stated<br>USA	1. MoP<br>Titanium: UHMWPE<br>28mm<br>Uncemented	21<br>0% bilateral	58 years (6)<br>70% female<br>100% OA<br>BMI 30 (4)	Revision, HHS, WOMAC, SF-12, UCLA activity score<br>6, 12, 24 months
	2. MoP<br>Titanium: HXLPE<br>28mm<br>Uncemented	25<br>4.2% bilateral	58 years (8)<br>44% female<br>87.5% OA<br>BMI 29 (5)	
Bal et al. 2005 (5-7)<br>1998 – 2001<br>USA	1. CoC<br>Alumina: Alumina<br>28mm<br>Uncemented	250<br>13% bilateral	55 years (14.7)<br>44.8% female<br>64% OA<br>BMI not reported	Revision, HHS<br>24 months
	2. CoP<br>Alumina: PE unspecified<br>28mm<br>Uncemented	250<br>12% bilateral	61 years (12.8)<br>53.2% female<br>73% OA<br>BMI not reported	
Bascarevic et al. 2010 (8)<br>2003 – 2008<br>Serbia	1. CoC<br>Alumina: Alumina<br>28mm<br>Uncemented	82<br>4.9% bilateral	53.9 years (7.1)<br>79% female<br>56% OA<br>BMI 26.7 (3.8)	Revision, HHS<br>2, 6, 12, 24, 36, 48, 60, 72 months
	2. MoP<br>Co-Cr: HXLPE<br>28-32mm<br>Uncemented	75<br>4% bilateral	55.6 years (6.5)<br>69% female<br>57% OA<br>BMI 27.8 (4.2)	
Beaupre et al. 2013 (9, 10)<br>1998 – 2003<br>Canada	1. CoC<br>Alumina: Alumina<br>32mm<br>Uncemented	48	51.3 years (6.9)<br>46% female<br>% OA not reported<br>BMI not reported	Revision, WOMAC, SF-12,<br>12, 60 months
	2. CoP<br>Alumina: HXLPE<br>28mm<br>Uncemented	44	53.6 years (6.5)<br>46% female<br>% OA not reported<br>BMI not reported	
Bjorgul et al. 2013* (11)<br>1999 – 2003<br>Norway	1. MoM<br>Co-Cr: Co-Cr<br>28mm<br>Cemented	129	63.3 years (40.7-72.9)<br>68% female<br>82% OA<br>BMI not reported	Revision, HHS<br>24, 60, 84 months
	2. MoP<br>Co-Cr-Mo: PE unspecified<br>28mm	137	62.8 (24.5-72.7)<br>69% female<br>80% OA	

	Cemented		BMI not reported	
	3: CoP<br>Alumina: PE unspecified<br>28mm<br>Cemented	131	63.9 years (30.6-73.8)<br>59% female<br>80% OA<br>BMI not reported	
Brodner et al.<br>2003 (12, 13)<br>1993 -<br>Austria	1. MoM<br>Co-Cr-Mo: Co-Cr-Mo<br>28mm<br>Uncemented	50	58.3 years (16-81)<br>62% female<br>%OA not reported<br>BMI not reported	Revision, HHS<br>3, 6, 12, 18, 24,<br>36, 48, 60 months
	2. CoP<br>Alumina: UHMWPE<br>28mm<br>Uncemented	50	62 (26-87)<br>78% female<br>%OA not reported<br>BMI not reported	
Calvert et al. 2009<br>(14)<br>2001 – 2003<br>New Zealand	1. MoP<br>Co-Cr: HXPLE<br>28mm<br>Hybrid	59<br>0% bilateral	62.5 years (46-75)<br>59.3% female<br>100% OA<br>BMI not reported	Revision, HHS,<br>UCLA activity<br>score<br>6, 12, 24, 36<br>months
	2. MoP<br>Co-Cr: Conventional PE<br>28mm<br>Hybrid	60<br>0% bilateral	61 years (48-100)<br>45% female<br>100% OA<br>BMI not reported	
D'Antonio et al.<br>2002 (15-26)<br>1996 – 1998<br>USA	1. CoC<br>Alumina: Alumina<br>28-32mm<br>Uncemented	349<br>9.7% bilateral	53 years<br>35% female<br>78.5% OA<br>BMI 28.7	Revision, HHS<br>2, 6, 12, 24, 36,<br>48, 60, 72, 84, 96,<br>108, 120 months
	2. MoP<br>Co-Cr: Conventional PE<br>26-32mm<br>Uncemented	165<br>2.5% bilateral	53 years<br>40% female<br>76% OA<br>BMI 28.7	
Dahlstrand et al.<br>2009 (27)<br>1998 – 2001<br>Sweden, USA	1. MoP<br>Co-Cr-Mo: PE unspecified<br>28mm<br>Cemented	26<br>0% bilateral	65 years (45-74)<br>%female<br>100% OA<br>BMI 26.7	HHS, SF-36<br>3, 6, 12, 24<br>months
	2. MoM<br>Co-Cr-Mo: Co-Cr-Mo<br>28mm<br>Cemented	28<br>0% bilateral	65 years (45-74)<br>46.4% female<br>100% OA<br>BMI 26.7	
Desmarchelier et<br>al. 2013 (28)<br>1999 - 2000<br>France	1. MoM<br>Co-Cr-Mo: Co-Cr-Mo<br>28mm<br>Uncemented	125<br>12.6% bilateral	63.7 years (12.7)<br>61.6% female<br>72.8% OA<br>BMI 25.4 (3.3)	Revision, HHS<br>Merle D'Aubigné<br>score<br>3, 12, 36, 60, 120<br>months
	2. CoC<br>Alumina: Alumina<br>28mm<br>Uncemented	125<br>7.8% bilateral	59.6 years (14.6)<br>45% female<br>68% OA<br>BMI 25.8 (3.9)	
Digas et al. 2003<br>(29-33)<br>1998 - 2001<br>Sweden	1. MoP<br>Co-Cr: HXLPE<br>28mm<br>Cemented	32<br>0% bilateral	Median 54 years<br>(35-68)<br>53.1% female<br>100% OA<br>BMI not reported	Revision, HHS<br>3, 6, 12, 24, 36,<br>60, 120 months
	2. MoP<br>Co-Cr: Conventional PE<br>28mm<br>Cemented	29<br>0% bilateral	Median 55 years (41-70)<br>48.3% female<br>100% OA<br>BMI not reported	
Dorr et al. 2004<br>(34)<br>Not stated<br>USA	1. MoM<br>Co-Cr-Mo: Co-Cr-Mo<br>28mm<br>Uncemented (cup)<br>Cemented or uncemented<br>stem	311	60.0 years<br>41.0% female<br>% OA not reported<br>BMI not reported	Revision, HHS<br>60 months
	2. CoP<br>Zirconia: Unspecified PE	304	59.5 years<br>% female	

	28mm<br>Uncemented (cup)<br>Cemented or uncemented stem		% OA not reported<br>BMI not reported	
Eggli et al. 2002 (35)<br>1986 - 1987<br>Switzerland	1. MoP<br>Co-Cr-Mo: UHMWPE<br>22mm<br>Cemented	49	Age not reported<br>% female not reported<br>% OA not reported<br>BMI not reported	None relevant (radiological outcomes only)<br>3, 6, 12, 24, 36, 48, 60, 72, 84, 96, 108 months
	2. MoP<br>Co-Cr-Mo: UHMWPE<br>32mm<br>Cemented	40	Age not reported<br>% female not reported<br>% OA not reported<br>BMI not reported	
Engh et al. 2006 (36, 37)<br>1999 - 2000<br>USA	1. MoP<br>Co-Cr: HXLPE<br>28mm<br>Uncemented	116	62.5 years (10.6, 26-87)<br>56 % female<br>85.3 % OA<br>BMI 28.6 (5.5)	Revision, HHS<br>60, 108, 120, 156 months
	2. MoP<br>Co-Cr: UHMWPE<br>28mm<br>Uncemented	114	62.0 years (11.1, 34-84)<br>50 % female<br>78.9 % OA<br>BMI 27.9 (5.1)	
Engh et al. 2009 (38, 39)<br>2003 - 2005<br>USA, Canada	1. MoP<br>Co-Cr: HXLPE<br>28mm<br>Uncemented	37<br>0% bilateral	61.6 years<br>29.7 % female<br>% OA not reported<br>BMI 29.9	Revision, HHS, WOMAC, SF-12<br>6, 12, 24, 60 months
	2. MoM<br>Co-Cr: Co-Cr<br>28mm<br>Uncemented	30<br>0% bilateral	62.2 years<br>60% female<br>% OA not reported<br>BMI 28.7	
	3: MoM<br>Co-Cr: Co-Cr<br>36mm<br>Uncemented	38<br>0% bilateral	63.4 years<br>28.9% female<br>% OA not reported<br>BMI 29.1	
Engh et al. 2015 (40)<br>2005 - 2006<br>USA, Canada	1. CoM<br>Zirconia/ Alumina: Co-Cr-Mo<br>28-36mm<br>Uncemented	194	59 years<br>43% female<br>83% OA<br>BMI 30	Revision, HHS<br>3, 12, 24, 48, 60, 72 months
	2. MoM<br>Co-Cr-Mo: Co-Cr-Mo<br>28-36mm<br>Uncemented	196	60 years<br>46% female<br>89% OA<br>BMI 30	
Garbuz et al. 2010* (41)<br>2005 - 2008<br>Canada	1. Resurfacing<br>Co-Cr: Co-Cr<br>40mm<br>Reverse hybrid	48<br>0% bilateral	51.5 years<br>10.4% female<br>100% OA<br>BMI 28.3	PAT-5D, WOMAC, SF-36, UCLA activity score<br>24 months
	2. MoM<br>Co-Cr-Mo: Co-Cr<br>40mm<br>Uncemented	56<br>0% bilateral	52 years<br>10.7% female<br>100% OA<br>BMI 28.2	
Garcia-Rey et al. 2008 (42, 43)<br>1999 - 2001<br>Spain	1. MoP<br>Co-Cr-Mo: HXLPE<br>28mm<br>Uncemented	45	62.5 years (47-78)<br>57.1% female<br>62.2% OA<br>BMI not reported	Revision, Merle D'Aubigné score<br>6, 12, 24, 36, 48, 60, 72, 84, 108, 120, 132, 144 months
	2. MoP<br>Stainless steel: UHMWPE<br>28mm<br>Uncemented	45	60.6 years (25-88)<br>53.7% female<br>71.1% OA<br>BMI not reported	
Garellick et al. 2000 (44-46)<br>1985 - 1989<br>Sweden	1. MoP<br>Stainless steel: PE unspecified<br>22mm<br>Cemented	206<br>9.3% bilateral	70 years (41-85)<br>70% female<br>57% OA<br>BMI not reported	Revision, HHS, Pain score, Satisfaction<br>12, 36, 72, 120, 132 months

	2. MoP<br>Co-Cr: PE unspecified<br>32mm<br>Cemented	204<br>9.3% bilateral	71 years (40-86)<br>64% female<br>63% OA<br>BMI not reported	
Gauthier et al.<br>2013 (47)<br>2007 - 2010<br>Canada	1. MoM<br>Co-Cr-Mo: Co-Cr-Mo<br>36-54<br>Uncemented	25<br>0% bilateral	60.2 years (7.2)<br>44% female<br>% OA not reported<br>BMI 30.8 (5.6)	Revision, HHS,<br>WOMAC, UCLA<br>activity score,<br>RAND-36<br>3, 6, 12, 24<br>months
	2. MoP<br>Co-Cr: HXLPE<br>28-32mm<br>Uncemented	25<br>0% bilateral	63 years (5.5)<br>54 % female<br>% OA not reported<br>BMI 29.2 (5.0)	
Geerdink et al.<br>2006 (48)<br>Not stated<br>The Netherlands	1. MoP<br>Co-Cr: Conventional PE<br>28mm<br>Uncemented (cup)	67	63 years (37-72)<br>% female not reported<br>94.4 % OA<br>BMI 27	Revision<br>2, 12, 24, 36, 60<br>months
	2. MoP<br>Co-Cr: MXLPE<br>28mm<br>Uncemented (cup)	66	64 years (45-74)<br>% female not reported<br>95.6 % OA<br>BMI 28	
Geerdink et al.<br>2009 (49)<br>Not stated<br>The Netherlands	1. MoP<br>Co-Cr: Conventional PE<br>28mm<br>Uncemented	26	64 years (54-72)<br>43.4% female<br>% OA not reported<br>BMI 28	Revision, HHS<br>2, 12, 24, 36, 60,<br>96 months
	2. MoP<br>Co-Cr: MXLPE<br>28mm<br>Uncemented	22	64 years (48-74)<br>35.3% female<br>% OA not reported<br>BMI 28	
Girard et al. 2006<br>(50-58)<br>2003 - 2006<br>Canada	1. Resurfacing<br>Co-Cr-Mo: Co-Cr-Mo<br>40mm<br>Hybrid	104<br>5.4% bilateral	49.2 years (9.0, 23-64)<br>37% female<br>80% OA<br>BMI 27 (5.3)	Revision,<br>WOMAC, Merle<br>D'Aubigné score,<br>UCLA activity<br>score<br>3, 6, 12, 24, 60,<br>96, 108, 120<br>months
	2. MoM<br>Co-Cr-Mo: Co-Cr-Mo<br>28mm<br>Uncemented	99<br>5.6% bilateral	51.0 years (8.6, 24-65)<br>32% female<br>80% OA<br>BMI 30 (6.8)	
Glyn-Jones et al.<br>2008 (59-62)<br>2002 - 2003<br>UK	1. MoP<br>Co-Cr: UHMWPE<br>28mm<br>Hybrid	27<br>0% bilateral	67 years (51-76)<br>50% female<br>100% OA<br>BMI not reported	Revision, OHS<br>3, 6, 12, 24, 36,<br>60, 84, 120<br>months
	2. MoP<br>Co-Cr: HXLPE<br>28mm<br>Hybrid	27<br>0% bilateral	68 years (52-76)<br>50% female<br>100% OA<br>BMI not reported	
Grubl et al. 2006*<br>(63)<br>2000 - 2002<br>Austria	1. CoC<br>Alumina: Alumina<br>28mm<br>Uncemented	15<br>0% bilateral	58.2 years (32-81)<br>53.3% female<br>92.9% OA<br>BMI not reported	HHS, UCLA<br>activity score<br>12 months
	2. MoM<br>Co-Cr-Mo: Co-Cr-Mo<br>28mm<br>Uncemented	13<br>0% bilateral	66.8 years (56-78)<br>76.9% female<br>92.9% OA<br>BMI not reported	
Haddad et al.<br>2015 (64, 65)<br>1999 – 2002<br>UK	1. Resurfacing<br>Co-Cr: Co-Cr<br>38-58mm<br>Hybrid	11	47.8 years (29-55)<br>25% female<br>% OA not reported<br>BMI not reported	HHS, WOMAC,<br>OHS, EQ5D, UCLA<br>activity score,<br>University College<br>Hospital<br>functional score<br>12, 60, 120<br>months
	2. MoP<br>Co-Cr: PE unspecified<br>32mm<br>Uncemented	13	48.2 years (31-55)<br>27.5% female<br>% OA not reported<br>BMI not reported	
Hailer et al. 2011<br>(66)	1. MoP<br>Co-Cr-Mo: HXLPE	44	Age not reported<br>% female not reported	HHS

1998 – 2001<br>Sweden	28mm<br>Cemented		% OA not reported<br>BMI not reported	3, 6, 12, 24, 72<br>months
	2. MoM<br>Co-Cr-Mo: Co-Cr-Mo<br>28mm<br>Cemented	41	Age not reported<br>% female not reported<br>% OA not reported<br>BMI not reported	
Hamilton et al.<br>2010 (67)<br>2003 –<br>USA, Canada	1. CoC<br>Alumina: Alumina<br>28mm<br>Uncemented	177	56.4 years<br>49% female<br>89% OA<br>BMI not reported	Revision, HHS<br>24 months
	2. CoP<br>Alumina: MXLPE<br>28mm<br>Uncemented	87	57.3 years<br>46% female<br>90% OA<br>BMI not reported	
Hanna et al. 2012<br>(68)<br>2007 – 2009<br>UK	1. MoP<br>Co-Cr: UHMWPE<br>28-32mm<br>Cemented	22	72 years (66-80)<br>77% female<br>100% OA<br>BMI 28.7	Revision, HHS,<br>OHS, SF-36<br>12, 24, 36 months
	2. MoM<br>Co-Cr: Co-Cr<br>44-54mm<br>Hybrid	27	68 years (60-76)<br>78% female<br>100% OA<br>BMI 28.1	
Howie et al. 2005<br>(69)<br>1993 – 1995<br>Australia	1. Resurfacing<br>Co-Cr: Metal unspec<br>40mm<br>Cemented	11<br>0% bilateral	Median 46 years (16-55)<br>45% female<br>64% OA<br>BMI not reported	Revision, HHS,<br>HHPS<br>3, 6, 12, 60, 120<br>months
	2. MoP<br>Stainless steel: UHMWPE<br>26mm<br>Cemented	13<br>0% bilateral	Median 50 years (22-54)<br>31% female<br>62% OA<br>BMI not reported	
Howie et al. 2012<br>(70)<br>2001 – 2007<br>Australia, UK	1. MoP<br>Unspecified metal: HXLPE<br>36mm<br>Hybrid	273<br>0% bilateral	72.3 years (5.9, 59-93)<br>56.0% female<br>96.3% OA<br>BMI 28.0 (5.9)	Revision<br>12 months
	2. MoP<br>Unspecified metal: HXLPE<br>28mm<br>Hybrid	284<br>0% bilateral	72.3 years (6.9, 60-92)<br>61.3% female<br>95.4% OA<br>BMI 28.4 (5.2)	
Jacobs et al. 2004<br>(71)<br>1997 – 2000<br>USA	1. MoM<br>Co-Cr: Unspecified metal<br>28mm<br>Uncemented	118<br>0% bilateral	53.3 years (18-75)<br>51.6% female<br>61.1% OA<br>BMI not reported	Revision, HHS<br>2, 6, 12, 24, 36,<br>48, 60, 72 months
	2. MoP<br>Co-Cr: MXLPE<br>28mm<br>Uncemented	118<br>0% bilateral	55.7 years (31-75)<br>32.9% female<br>71.1% OA<br>BMI not reported	
Jassim et al. 2015<br>(72-74)<br>2005 – 2008<br>UK	1. MoP<br>Co-Cr: HXLPE<br>32mm<br>Uncemented	133	61 years (51-77)<br>68.4% female<br>% OA not reported<br>BMI not reported	Revision,<br>WOMAC, SF-36,<br>VAS pain<br>6, 12, 24, 36, 48,<br>60 months
	2. CoP<br>Zirconia: HXLPE<br>32mm<br>Uncemented	135	63 years (47-75)<br>55.6% female<br>% OA not reported<br>BMI not reported	
	3. CoP<br>Zirconia: UHMWPE<br>32mm<br>Uncemented	133	63 years (53-73)<br>62.4% female<br>% OA not reported<br>BMI not reported	
Jensen et al.<br>2011* (75-78)<br>2007 - 2009<br>Denmark	1. Resurfacing<br>Co-Cr-Mo: Co-Cr-Mo<br>47-57mm<br>Hybrid	21	57 years (5.1, 45-65)<br>39 % female<br>% OA not reported<br>BMI 27.3 (5.1)	Revision<br>2, 6, 12, 24<br>months

	2. CoP<br>Alumina: Unspecified PE<br>28mm<br>Uncemented	22	55 years (6.2, 44-64)<br>16% female<br>100% OA<br>BMI 28.4 (2.8)	
Kadar et al. 2011* (79-81)<br>2004 – 2007<br>Norway	1. MoP<br>Stainless steel: UHMWPE<br>22mm<br>Cemented	30<br>0% bilateral	70 years (6.1)<br>67% female<br>93% OA<br>BMI not reported	Revision, HHS<br>3, 12, 24 months
	2. MoP<br>Co-Cr: UHMWPE<br>28mm<br>Cemented	30<br>0% bilateral	69 years (5.9)<br>67% female<br>87% OA<br>BMI not reported	
	3. CoP<br>Zirconium: UHMWPE<br>28mm<br>Cemented	30<br>0% bilateral	69 years (6.7)<br>77% female<br>87% OA<br>BMI not reported	
	4. MoP<br>Co-Cr: HXLPE<br>28mm<br>Cemented	30<br>0% bilateral	70 years (5.3)<br>67% female<br>73% OA<br>BMI not reported	
	5. CoP<br>Zirconium: HXLPE<br>28mm<br>Cemented	30<br>0% bilateral	70 years (5.4)<br>73% female<br>90% OA<br>BMI not reported	
Kelley et al. 1998 (82)<br>1995 – 1996<br>USA	1. MoP<br>Unspecified metal: Unspecified PE<br>22mm<br>Uncemented cup, stem not stated	14	Age not reported<br>% female not reported<br>% OA not reported<br>BMI not reported	Revision<br>12 months
	2. MoP<br>Unspecified metal: Unspecified PE<br>28mm<br>Uncemented cup, stem not stated	17	Age not reported<br>% female not reported<br>% OA not reported<br>BMI not reported	
Kraay et al. 2006 (83)<br>- 2001<br>USA	1. CoP<br>Zirconia: UHMWPE<br>28mm<br>Hybrid	30	69.5 years<br>74.1% female<br>% OA not reported<br>BMI not reported	Revision, HHS<br>48 months
	2. MoP<br>Co-Cr-Mo: UHMWPE<br>28 mm<br>Hybrid	30	68.9 years<br>63.3% female<br>% OA not reported<br>BMI not reported	
Langlois et al. 2015 (84)<br>2000-2002<br>France	1. MoP<br>Co-Cr: HXLPE<br>22mm<br>Cemented	50<br>0% bilateral	66.4 years (SD 12.9)<br>55% female<br>100% OA<br>BMI 24.4 (3.7)	Revision, Merle D'Aubigné Score<br>Minimum 96 months
	2. MoP<br>Co-Cr: Conventional PE<br>22mm<br>Cemented	50<br>0% bilateral		
Lavigne et al. 2010* (85)<br>2006 – 2007<br>Canada	1. Resurfacing<br>Unspecified metal: Unspecified metal<br>40mm<br>Hybrid	24	49.6 years (38-63)<br>41.7% female<br>76% OA<br>BMI 27.9	Revision, WOMAC, SF-36, Merle D'Aubigné Scale, UCLA activity score<br>3, 6, 12, 18 months
	2. MoM<br>Unspecified metal: Unspecified metal<br>40mm<br>Uncemented	24	49.8 years (33-62)<br>37.5% female<br>80% OA<br>BMI 27.8	

Lewis et al. 2008 (86)<br>Not stated<br>Canada	1. CoP<br>Zirconium: Standard PE or HXLPE<br>Not stated<br>Uncemented	50	51 years (10.8, 22-74)<br>48% female<br>76% OA<br>BMI not reported	Revision, HHS<br>WOMAC, SF-12<br>3, 12, 24 months
	2. MoP<br>Co-Cr: Standard PE or HXLPE<br>Not stated<br>Uncemented	50	51 years (11.0, 19-76)<br>48% female<br>76% OA<br>BMI not reported	
Lindalen et al. 2015 (87)<br>2009-2010<br>Norway	1. CoP<br>Zirconia/ alumina: HXLPE (vitamin E)<br>32mm<br>Uncemented	25<br>0% bilateral	62 years (3.8)<br>84% female<br>100% OA<br>BMI 28 (6.4)	HHS, OHS, UCLA activity score<br>24 months
	2. CoP<br>Zirconia/ alumina: HXLPE (vitamin E)<br>36mm<br>Uncemented	25<br>4% bilateral	61 years (3.8)<br>56% female<br>100% OA<br>BMI 28 (5.1)	
Lombardi et al. 2001 (88, 89)<br>1995 – 1999<br>USA	1. MoM<br>Co-Cr-Mo: Co-Cr-Mo<br>28mm<br>Uncemented	97<br>2.8% bilateral	49.3 years (1.1, 26-73)<br>25.6% female<br>74.4% OA<br>BMI 29.1 (0.61)	Revision, HHS<br>24, 60 months
	2. MoP<br>Co-Cr-Mo: UHMWPE<br>30mm<br>Uncemented	97<br>2.8% bilateral	48.9 years (1.4, 18-77)<br>23.6% female<br>72.2% OA<br>BMI 28.7 (0.59)	
Lombardi et al. 2010 (90)<br>2000 –<br>USA	1. CoC<br>Alumina: Alumina<br>28-32mm<br>Uncemented	64	57 years (33-76)<br>45% female<br>86% OA<br>BMI 29.9	Revision, HHS<br>6, 12, 24, 36, 48, 60, 72, 84, 96, 108 months
	2. CoP<br>Zirconia: HXLPE<br>28-32mm<br>Uncemented	45	60 years (35-79)<br>47% female<br>84% OA<br>BMI 28.8	
MacDonald et al. 2003* (91)<br>1998 – 1999<br>Canada	1. MoM<br>Co-Cr-Mo: Co-Cr-Mo<br>28mm<br>Uncemented	23	Age not reported<br>% female not reported<br>% OA not reported<br>BMI not reported	Revision, HHS, WOMAC, SF-12<br>2, 3, 6, 12, 24, 36, 48 months
	2. MoP<br>Co-Cr-Mo: UHMWPE<br>28mm<br>Uncemented	18	Age not reported<br>% female not reported<br>% OA not reported<br>BMI not reported	
Malviya et al. 2011 (92)<br>Check<br>UK	1. MoM<br>Co-Cr: Unspecified metal<br>38-54mm<br>Hybrid	50<br>5% bilateral	63.9 years (42-82)<br>62% female<br>% OA not reported<br>BMI 28.6	Revision, HHS, WOMAC, SF-36, HOOS<br>3, 12, 24 months
	2. MoP<br>Unspecified metal: Unspecified PE<br>28mm<br>Hybrid	50<br>5% bilateral	64.9 years (42-85)<br>54% female<br>% OA not reported<br>BMI 29.4	
Marston et al. 1996 (93, 94)<br>1982 – 1987<br>UK	1. MoP<br>Unspecified metal: Unspecified PE<br>29mm<br>Cemented	213	Age not reported<br>% female not reported<br>88.7% OA<br>BMI not reported	Revision, HHS<br>12, 24, 36, 48, 60, 72, 84, 96, 108, 120 months
	2. MoP<br>Unspecified metal: Unspecified PE<br>22mm	200	Age not reported<br>% female not reported<br>89.5% OA<br>BMI not reported	

	Cemented			
Martell et al.<br>2003 (95)<br>1999 – 2001<br>USA	1. MoP<br>Co-Cr: HXLPE<br>28mm<br>Uncemented	24	60 years (28-76)<br>% female not reported<br>% OA not reported<br>BMI 30.6	Revision<br>3, 12, 24, 36, 48 months
	2. MoP<br>Co-Cr: UHMWPE<br>28mm<br>Uncemented	22	55 years (28-75)<br>% female not reported<br>% OA not reported<br>BMI 27.6	
McCalden et al.<br>2009* (96)<br>1999 – 2001<br>Canada	1. MoP<br>Co-Cr: HXLPE<br>28mm<br>Hybrid	50	72.3 years (56-79)<br>66% female<br>100% OA<br>BMI 29.7	Revision, HHS, WOMAC, SF-12<br>2, 3, 12, 24, 36, 48, 60 months
	2. MoP<br>Co-Cr: Conventional PE<br>28mm<br>Hybrid	50	72.6 years (56-79)<br>72% female<br>100% OA<br>BMI 29.7	
Morison et al.<br>2014 (97)<br>2004 – 2007<br>Canada, UK	1. MoP<br>Co-Cr: UHMWPE<br>28mm<br>Uncemented	21	50.6 years (31.7-67.5)<br>47.6% female<br>% OA not reported<br>BMI 30.3	Revision, HHS, WOMAC, SF-12<br>3, 12, 24, 60 months
	2. MoP<br>Co-Cr: HXLPE<br>28mm<br>Uncemented	24	53.7 years (22.8-66.0)<br>47.8% female<br>% OA not reported<br>BMI 27.9	
	3. CoP<br>Zirconium: UHMWPE<br>28mm<br>Uncemented	22	52.4 years (39.8-64.8)<br>36.4% female<br>% OA not reported<br>BMI 27.1	
	4. CoP<br>Zirconium: HXLPE<br>28mm<br>Uncemented	24	51.2 years (22.6-63.9)<br>54.5% female<br>% OA not reported<br>BMI 29.3	
Mutimer et al.<br>2010 (98)<br>2001 – 2003<br>New Zealand	1. MoP<br>Co-Cr: HXLPE<br>28mm<br>Hybrid	61	62 years (46-75)<br>36.4% female<br>100% OA<br>BMI not reported	Revision<br>2, 6, 12, 24, 36, 48, 60, 72 months
	2. MoP<br>Co-Cr: Conventional PE<br>28mm<br>Hybrid	61	61 years (48-75)<br>53.0% female<br>100% OA<br>BMI not reported	
Nakahara et al.<br>2010 (99)<br>2000 – 2001<br>Japan	1. CoP<br>Zirconia: HXLPE<br>26mm<br>Uncemented	51<br>6.8% bilateral	57.5 years (9.5, 27-76)<br>80.9% female<br>89.4% OA<br>BMI 23.5 (3.8)	Revision, Merle D'Aubigné score, UCLA activity score<br>3, 6, 12, 24, 36, 48, 60, 72, 84 months
	2. MoP<br>Co-Cr: HXLPE<br>26mm<br>Uncemented	51<br>2.2% bilateral	56.9 years (10.5, 27-75)<br>87.2% female<br>87.2% OA<br>BMI 23.5 (4.2)	
Nikolaou et al.<br>2012 (100)<br>2003 – 2005<br>Canada	1. MoP<br>Co-Cr: UHMWPE<br>28mm<br>Uncemented	36	52.6 years (20-64)<br>50.0% female<br>63.9% OA<br>BMI 28.7	Revision, HHS, WOMAC, SF-12<br>3, 12, 24, 36, 48, 60 months
	2. MoP<br>Co-Cr: HXLPE<br>28mm<br>Uncemented	32	55.1 years (41-64)<br>43.8% female<br>59.4% OA<br>BMI 32.6	
	3. CoC<br>Unspecified ceramic: Unspecified ceramic<br>28mm<br>Uncemented	34	52.0 years (19-64)<br>50.0% female<br>73.5% OA<br>BMI 28.2	

Ochs et al. 2007 (101)<br>1997 – 1999<br>Germany	1. CoC<br>Alumina: Unspecified ceramic<br>28mm<br>Uncemented	35	56.0 years (7.6)<br>31.8% female<br>81.8% OA<br>BMI not reported	Revision, HHS, Hannover Function Score<br>3, 12, 24, 60, 96, 108 months
	2. CoP<br>Alumina: Conventional PE<br>28mm<br>Uncemented	31	61.5 years (7.0)<br>33.3% female<br>80.9% OA<br>BMI not reported	
Pabinger et al. 2003 (102)<br>1993 – 1995<br>Austria	1. MoM<br>Co-Cr-Mo: Co-Cr-Mo<br>30mm<br>Cemented	32<br>3.2% bilateral	50-70 years<br>38.7% female<br>94% OA<br>BMI not reported	Revision<br>3, 6, 12, 24, 36, 48 months
	2. CoP<br>Unspecified ceramic: UHMWPE (vitamin E)<br>30mm<br>Cemented	29<br>3.6% bilateral	56-70 years<br>42.9% female<br>97% OA<br>BMI not reported	
Penny et al. 2013* (75-77, 103) 2007 – 2009<br>Denmark	1. MoP<br>Co-Cr: UHMWPE or HXLPE<br>28mm<br>Uncemented	15	58 years (48-66)<br>46.7% female<br>100% OA<br>BMI 27	Revision, HHS, WOMAC, EQ-5D, UCLA activity score<br>6, 12, 24 months
	2. MoM<br>Co-Cr: Co-Cr<br>47-56mm<br>Uncemented	18	63 years (54-64)<br>47.0% female<br>100% OA<br>BMI 28	
Pitto et al. 2003 (104, 105)<br>Not stated<br>New Zealand, Germany	1. CoP<br>Alumina: UHMWPE<br>28mm<br>Uncemented cup and uncemented or cemented stem	27	Age not reported<br>% female not reported<br>% OA not reported<br>BMI not reported	HHS<br>3, 6, 12, 24 months
	2. CoC<br>Alumina: Alumina<br>28mm<br>Uncemented cup and uncemented or cemented stem	23	Age not reported<br>% female not reported<br>% OA not reported<br>BMI not reported	
Pitto et al. 2002 (106)<br>Not stated<br>New Zealand	1. CoC<br>Zirconia: Alumina<br>32mm<br>Hybrid	5	59 years (53-66)<br>80% female<br>% OA not reported<br>BMI 24.0	Revision, HHS<br>60 months
	2. CoC<br>Alumina: Alumina<br>32mm<br>Hybrid	5	57 years (56-67)<br>80% female<br>% OA not reported<br>BMI 25.1	
Poggie et al. 2007 (107)<br>1999 – 2003<br>USA	1. CoC<br>Alumina: Alumina<br>28mm<br>Uncemented	315<br>11.7% bilateral	54 years (12)<br>45% female<br>77% OA<br>BMI 29 (4.6)	Revision, HHS, SF-12<br>12, 24, 72 months
	2. CoP<br>Alumina: UHMWPE<br>28mm<br>Uncemented	157<br>6.8% bilateral		
Politi et al. 2013 (108)<br>2005 – 2010<br>USA	1. CoC<br>Alumina: Alumina<br>28mm<br>Uncemented	177	56.4 years (10.6)<br>49.2% female<br>%OA not reported<br>BMI not reported	HHS<br>6, 12, 24 months
	2. CoP<br>Alumina: HXLPE<br>28mm<br>Uncemented	87	57.3 years (10.3)<br>54.0% female<br>% OA not reported<br>BMI not reported	

Salemyr et al. 2015 (109)<br>2009<br>Sweden	1. MoP<br>Co-Cr: UHMWPE (vitamin E)<br>32mm<br>Uncemented	25<br>0% bilateral	62 years (6)<br>56.0% female<br>100% OA<br>BMI 28 (4)	Revision, HHS, EQ-5D<br>3, 6, 12, 24 months
	2. MoP<br>Co-Cr: HXLPE<br>32mm<br>Uncemented	26<br>0% bilateral	62 years (5)<br>57.7% female<br>100% OA<br>BMI 27 (4)	
Schouten et al. 2012 (110)<br>2009 – 2010<br>New Zealand	1. CoM<br>Zirconia/ alumina: Co-Cr-Mo<br>28-36mm<br>Uncemented	42<br>0% bilateral	61.5 years (42-75)<br>45% female<br>% OA not reported<br>BMI 29	Revision, WOMAC, OHS, UCLA activity score, VAS pain<br>6, 12 months
	2. MoM<br>Co-Cr-Mo: Co-Cr-Mo<br>28-36mm<br>Uncemented	39<br>0% bilateral	63.8 years (44-76)<br>36% female<br>% OA not reported<br>BMI 29	
Shareghi et al. 2015 (111)<br>2008-2010<br>Sweden	1. MoP<br>Co-Cr: UHMWPE (vitamin E)<br>32mm<br>Uncemented	38	Median 58 years (32-75)<br>42.1% female<br>100% OA<br>BMI 25	Revision, HHS, HPS pain score, UCLA activity score<br>3, 12, 24 months
	2. MoP<br>Co-Cr: HXLPE<br>32mm<br>Uncemented	32	Median 58 years (36-67)<br>53.1% female<br>100% OA<br>BMI 27	
Smolders et al. 2011 (112-114)<br>2007 – 2010<br>The Netherlands	1. Resurfacing<br>Co-Cr: Co-Cr<br>49mm<br>Hybrid	42<br>13.2% bilateral	Median 58 years (24-65)<br>44.7% female<br>92.1% OA<br>BMI 26.0 (3.1)	Revision, HHS, OHS, SF-12, UCLA activity score, VAS satisfaction<br>6, 12, 24, 36, 60 months
	2. MoM<br>Co-Cr: Co-Cr<br>28mm<br>Uncemented	40<br>12.1% bilateral	Median 59 (37-65)<br>36.4% female<br>93.9% OA<br>BMI 28.0 (5.1)	
Tiusanen et al. 2013 (115)<br>Not stated<br>Finland	1. MoP<br>Co-Cr-Mo: Unspecified PE<br>30mm<br>Uncemented	46	62 years<br>50.0% female<br>80.4% OA<br>BMI not reported	HHS, OHS<br>12, 24 months
	2. MoM<br>Co-Cr-Mo: Co-Cr-Mo<br>30mm<br>Uncemented	46	60 years<br>47.8% female<br>71.7% OA<br>BMI not reported	
Triclot et al. 2007 (116)<br>2000 – 2001<br>France	1. CoP<br>Stainless steel: HXLPE<br>28mm<br>Cemented	49<br>0% bilateral	67.9 years (44.1-85.2)<br>48.5% female<br>100% OA<br>BMI 26.5	Revision, Merle D'Aubigné score<br>60 months
	2. MoP<br>Stainless steel: UHMWPE<br>28mm<br>Cemented	53<br>0% bilateral	70.1 years (44.8-82.6)<br>41.2% female<br>100% OA<br>BMI 26.4	
Venditolli et al. 2007 (117-119)<br>1996 – 2001<br>Canada	1. CoC<br>Alumina: Alumina<br>32mm<br>Hybrid	71<br>19.1% bilateral	54.9 years (12.5, 23-70)<br>57.7% female<br>62.0% OA<br>BMI 28.2	Revision, WOMAC, UCLA activity score, Merle D'Aubigné score<br>24, 84, 108, 132, 157+ months
	2. MoP<br>Stainless steel: UHMWPE<br>28mm<br>Hybrid	69<br>36.2% bilateral	56.8 years (10.7, 29-70)<br>44.9% female<br>62.3% OA<br>BMI 27.3	
von Schewelov et al. 2005 (120)<br>1996 – 1998	1. MoP<br>Stainless steel: UHMWPE<br>22mm	30	64 years (50-76)<br>54.4% female<br>100% OA	Revision, HHS<br>3, 6, 12, 24, 60 months

Sweden	Cemented		BMI not reported	
	2. MoP<br>Stainless steel: Hylamer UHMWPE<br>22 mm<br>Cemented	30		
	3. CoP<br>Zirconia: UHMWPE<br>22mm<br>Cemented	30		
	4. CoP<br>Zirconia: Hylamer UHMWPE<br>22mm<br>Cemented	30		
Weissinger et al. 2011 (121)<br>Not stated<br>Austria	1. MoM<br>Co-Cr-Mo: Co-Cr-Mo<br>28mm<br>Uncemented	42	Median 58.5 years (34.3-77.9)<br>66.7% female<br>88.1% OA<br>BMI 28.1	HHS, UCLA activity score<br>24 months
	2. CoC<br>Alumina: Alumina<br>28mm<br>Uncemented	38	Median 64.8 years (44.1-81.8)<br>64.4% female<br>89.5% OA<br>BMI 27.4	
Williams et al. 2007* (122-124)<br>2004 –<br>South Africa	1. CoM<br>Zirconia/ Alumina: Co-Cr-Mo<br>28mm<br>Uncemented	7	47.7 years (17.4-66.7)<br>71.4% female<br>% OA not reported<br>BMI not reported	None relevant (metal ion levels)<br>6, 12, 24, 48, 108 months
	2. MoM<br>Co-Cr-Mo: Co-Cr-Mo<br>28mm<br>Uncemented	7	47.1 years (31.6-58.9)<br>85.7% female<br>% OA not reported<br>BMI not reported	
	3. CoP<br>Zirconia/ Alumina: HXLPE<br>28mm<br>Uncemented	10	51.3 years (32.0-66.2)<br>60.0% female<br>%OA not reported<br>BMI not reported	
	4. CoC<br>Zirconia/ Alumina: Zirconia/ Alumina<br>28mm<br>Uncemented	7	47.8 years (32.5-57.9)<br>42.9% female<br>%OA not reported<br>BMI not reported	
Zagra et al. 2013 (125, 126)<br>2007 – 2009<br>Italy	1. CoP<br>Unspecified ceramic: HXLPE<br>28mm<br>Uncemented	20	64.4 years (53-69)<br>100% female<br>100% OA<br>BMI 27.5	Revision, HHS, 2, 4 months
	2. CoP<br>Unspecified ceramic: HXLPE<br>36mm<br>Uncemented	20	65.9 years (55-71)<br>100% female<br>100% OA<br>BMI 27.1	
	3. MoM<br>Unspecified metal: Unspecified metal<br>42-48mm<br>Uncemented	20	65.2 years (56-70)<br>100% female<br>100% OA<br>BMI 26.1	
Zaoui et al. 2015 (127)<br>2006<br>France	1. MoP<br>Stainless steel: UHMWPE<br>22mm<br>Cemented	25<br>0% bilateral	Median 64 years (21-72)<br>59% female<br>100% OA<br>BMI 26	Revision, Merle D’Aubigné score<br>72 months

	2. CoP<br>Zirconia: UHMWPE<br>22mm<br>Cemented	25<br>0% bilateral	Median 60 years (41-75)<br>54% female<br>100% OA<br>BMI 25	
	3. MoP<br>Stainless steel: HXLPE<br>22mm<br>Cemented	25<br>0% bilateral	Median 65 years (43-75)<br>56% female<br>100% OA<br>BMI 27	
	4. Zirconia: HXLPE<br>22mm<br>Cemented	25<br>0% bilateral	Median 58 years (41-75)<br>50% female<br>100% OA<br>BMI 26	
Zerahn et al. 2011<br>(128)<br>2001 – 2003<br>Denmark	1. CoP<br>Zirconia: UHMWPE<br>Not stated<br>Hybrid	76<br>0% bilateral	25.1-87.3 years<br>37.3% female<br>%OA not reported<br>BMI not reported	Revision, OHS<br>48 months
	2. MoM<br>Co-Cr-Mo: Co-Cr-Mo<br>Not stated<br>Hybrid	74<br>0% bilateral	34.8-94.2 years<br>62.0% female<br>%OA not reported<br>BMI not reported	
	3. CoP<br>Zirconia: UHMWPE<br>Not stated<br>Hybrid	73<br>0% bilateral	54.2-87.1 years<br>65.0% female<br>%OA not reported<br>BMI not reported	
	4. CoC<br>Alumina: Alumina<br>Not stated<br>Hybrid	76<br>0% bilateral	50.4-87.2 years<br>73.0% female<br>%OA not reported<br>BMI not reported	
Zhou et al. 2006<br>(129)<br>2001 – 2002<br>Australia	1. CoC<br>Alumina: Alumina<br>28mm<br>Hybrid	31<br>0% bilateral	66 years (46-87)<br>67.7% female<br>%OA not reported<br>BMI not reported	WOMAC, SF-36<br>24 months
	2. MoP<br>Co-Cr: HXLPE<br>28mm<br>Hybrid	30<br>0% bilateral	68 years (53-83)<br>46.7% female<br>%OA not reported<br>BMI not reported	
Zijlstra et al. 2009<br>(130, 131)<br>1998 – 1999<br>The Netherlands	1. MoM<br>Co-Cr-Mo: Co-Cr-Mo<br>28mm<br>Cemented	102<br>0% bilateral	72 years (7.0)<br>79.4% female<br>%OA not reported<br>BMI not reported	Revision, HHS<br>OHS<br>12, 24, 60, 120<br>months
	2. MoP<br>Co-Cr-Mo: UHMWPE<br>28mm<br>Cemented	98<br>0% bilateral	69 years (8.0)<br>79.6% female<br>%OA not reported<br>BMI not reported	
Zijlstra et al. 2011<br>(132, 133)<br>2006 – 2008<br>The Netherlands	1. MoM<br>Co-Cr: Co-Cr<br>42-56<br>Uncemented	50	60 years (5.0)<br>48.0% female<br>%OA not reported<br>BMI not reported	Revision, HHS,<br>OHS<br>12 months
	2. MoP<br>Co-Cr: UHMWPE<br>28mm<br>Uncemented	54	61 years (5.0)<br>48.0% female<br>%OA not reported<br>BMI not reported	

Studies whose authors provided further data and information about study conduct are marked with an asterisk. Bearing combinations (Femoral head bearing surface on acetabular bearing surface): MoP metal-on-plastic, MoM metal-on-metal, CoC ceramic-on-ceramic, CoP ceramic-on-plastic, CoM ceramic-on-metal; PE polyethylene, UHMWPE ultra-high molecular weight polyethylene, HXLPE highly cross-linked polyethylene, MXLPE moderately cross linked polyethylene, Co-Cr cobalt-chrome, Co-Cr-Mo cobalt-chrome-molybdenum. OA osteoarthritis, BMI body mass index.

OHS Oxford Hip Score, HHS Harris Hip Score, HHPS Harris Hip Pain Score, WOMAC Western Ontario and McMaster Universities Arthritis Index, EQ-5D EuroQol five dimensions questionnaire, PAT-5D Paper Adaptive Test in 5 Domains, SF-36 Short Form 36 items, SF-12 Short Form 12 items, HOOS Hip Disability and Osteoarthritis Outcome Score

References of the included trials

1. Amanatullah DF, Landa J, Strauss EJ, Garino JP, Kim SH, Cesare PE. Comparison of surgical outcomes and implant wear between ceramic-ceramic and ceramic-polyethylene articulations in total hip arthroplasty. Journal of arthroplasty [Internet]. 2011; 26(6 Suppl):[72-7 pp.]. Available from: http://onlinelibrary.wiley.com/o/cochrane/clcentral/articles/259/CN-00811259/frame.html http://ac.els-cdn.com/S0883540311002166/1-s2.0-S0883540311002166-main.pdf?_tid=4726eede-b2bd-11e4-a362-00000aab0f6b&acdnat=1423748854_7bd66b3a5cd479afd021eb50b0f95a36.
2. Jarit G, Wasserman BR, Garino JP, Walsh M, Walter NE, DiCesare PE, Jiranek WA, Malvitz TA. Total hip arthroplasty with a ceramic-ceramic vs. ceramic-polyethylene bearing [abstract]. American Academy of Orthopaedic Surgeons Annual Meeting; 2006 Mar 22-26; Chicago (IL) www3aaosorg/education/anmeet/anmt2006/podium/ppr06_22cfm (accessed 26/03/08) [Internet]. 2006. Available from: http://onlinelibrary.wiley.com/o/cochrane/clcentral/articles/892/CN-00635892/frame.html.
3. Ando W, Yamamoto K, Atsumi T, Tamaoki S, Oinuma K, Shiratsuchi H, Tokunaga H, Inaba Y, Kobayashi N, Aihara M, Ohzono K. Comparison between component designs with different femoral head size in metal-on-metal total hip arthroplasty; multicenter randomized prospective study. J. 2015;12(4):228-36.
4. Ayers DC, Hays PL, Drew JM, Eskander MS, Osuch D, Bragdon CR. Two-year radiostereometric analysis evaluation of femoral head penetration in a challenging population of young total hip arthroplasty patients. Journal of arthroplasty [Internet]. 2009; 24(6 Suppl):[9-14 pp.]. Available from: http://onlinelibrary.wiley.com/o/cochrane/clcentral/articles/452/CN-00733452/frame.html http://ac.els-cdn.com/S0883540309002083/1-s2.0-S0883540309002083-main.pdf?_tid=a373ab0a-b2bd-11e4-990c-00000aab0f27&acdnat=1423749008_9722d702af37b287ff66c77231e8ba28.
5. Bal SB, Aleto TJ, Garino JP, Toni A, Hendricks KJ. Ceramic-on-ceramic versus ceramic-on-polyethylene bearings in total hip arthroplasty: Results of a multicenter prospective randomized study and update of modern ceramic total hip trials in the United States. Hip Int. 2005;15(3):129-35.
6. Newman MA. Cementless Alumina/Alumina ceramic total hip arthroplasty. A prospective randomized study with 12 to 38 month follow up. The Journal of Bone and Joint Surgery (Proceedings) [Internet]. 2004; 86-b(Supp_iv):[393 p.]. Available from: http://www.bjjprocs.boneandjoint.org.uk/content/86-B/SUPP_IV/393.1.short.
7. Friedman R, Longo J, Cherry K, Newman M, Jessup D, Bal S. Prospective randomized study comparing ceramic/ceramic and ceramic/PE bearing surfaces in THA. Journal of Bone & Joint Surgery - British Volume. 2008;90-B(Supp I):143-4.
8. Bascarevic Z, Vukasinovic Z, Slavkovic N, Dulic B, Trajkovic G, Bascarevic V, Timotijevic S. Alumina-on-alumina ceramic versus metal-on-highly cross-linked polyethylene bearings in total hip arthroplasty: a comparative study. International orthopaedics [Internet]. 2010; 34(8):[1129-35 pp.]. Available from: http://onlinelibrary.wiley.com/o/cochrane/clcentral/articles/070/CN-00778070/frame.html http://www.ncbi.nlm.nih.gov/pmc/articles/PMC2989085/pdf/264_2009_Article_899.pdf.
9. Beaupre LA, Manolescu A, Johnston DW. A randomized trial of ceramic-on-ceramic bearing versus ceramic-on-crossfire-polyethylene bearing in total hip arthroplasty: five-year outcomes. Journal of arthroplasty [Internet]. 2013; 28(3):[485-9 pp.]. Available from: http://onlinelibrary.wiley.com/o/cochrane/clcentral/articles/892/CN-00966892/frame.html http://ac.els-cdn.com/S0883540312005554/1-s2.0-S0883540312005554-main.pdf?_tid=920e29ee-b2bd-11e4-a6fc-00000aab0f26&acdnat=1423748979_cc5b8294cd0509746d729b916651e669.

10. Beaupre LA, Al-Houkail A, Johnston DW. A Randomized Trial Comparing Ceramic-on-Ceramic Bearing vs Ceramic-on-Crossfire-Polyethylene Bearing Surfaces in Total Hip Arthroplasty. Journal of Arthroplasty. 2016;31(6):1240-5.
11. Bjorgul K, Novicoff WN, Andersen ST, Ahlund OR, Bunes A, Wiig M, Brevig K. High rate of revision and a high incidence of radiolucent lines around Metasul metal-on-metal total hip replacements: results from a randomised controlled trial of three bearings after seven years. Bone & Joint Journal. 2013;95-B(7):881-6.
12. Brodner W, Bitzan P, Meisinger V, Kaider A, Gottsauner-Wolf F, Kotz R. Serum cobalt levels after metal-on-metal total hip arthroplasty. Journal of Bone & Joint Surgery - American Volume. 2003;85-A(11):2168-73.
13. Brodner W, Bitzan P, Meisinger V, Kaider A, Gottsauner-Wolf F, Kotz R. Elevated serum cobalt with metal-on-metal articulating surfaces.[Erratum appears in J Bone Joint Surg Br 1997 Sep;79(5):885]. Journal of Bone & Joint Surgery - British Volume. 1997;79(2):316-21.
14. Calvert GT, Devane PA, Fielden J, Adams K, Horne JG. A double-blind, prospective, randomized controlled trial comparing highly cross-linked and conventional polyethylene in primary total hip arthroplasty. Journal of Arthroplasty. 2009;24(4):505-10.
15. D'Antonio J, Capello W, Manley M, Bierbaum B. New experience with alumina-on-alumina ceramic bearings for total hip arthroplasty. Journal of Arthroplasty. 2002;17(4):390-7.
16. Bierbaum BE, Nairus J, Kuesis D, Morrison JC, Ward D. Ceramic-on-ceramic bearings in total hip arthroplasty. Clinical Orthopaedics & Related Research. 2002(405):158-63.
17. Capello WN, Dantonio JA, Feinberg JR, Manley MT. Alternative bearing surfaces: alumina ceramic bearings for total hip arthroplasty. Instructional Course Lectures. 2005;54:171-6.
18. Capello WN, D'Antonio JA, Manley MT, Feinberg JR. Arc-deposited hydroxyapatite-coated cups: results at four to seven years. Clinical orthopaedics and related research [Internet]. 2005; 441:[305-12 pp.]. Available from: http://onlinelibrary.wiley.com/o/cochrane/clcentral/articles/334/CN-00532334/frame.html http://graphics.tx.ovid.com/ovftpdfs/FPDDNCDCIHHJMB00/fs046/ovft/live/gv023/00003086/00003086-200512000-00046.pdf.
19. Capello WN, D'Antonio JA, Feinberg JR, Manley MT, Naughton M. Ceramic-on-ceramic total hip arthroplasty: update. Journal of arthroplasty [Internet]. 2008; 23(7 Suppl):[39-43 pp.]. Available from: http://onlinelibrary.wiley.com/o/cochrane/clcentral/articles/433/CN-00665433/frame.html http://ac.els-cdn.com/S0883540308005196/1-s2.0-S0883540308005196-main.pdf?_tid=3cdde39c-b2bd-11e4-b8e3-00000aab0f01&acdnat=1423748836_d5c02a7bb2fb3720308a1a5d9366310f.
20. D'Antonio J, Capello W, Manley M, Bierbaum B. Alumina ceramic bearings for total hip arthroplasty. Seminars in Arthroplasty. 2003;14(2):104-12.
21. D'Antonio JA, Capello WN, Manley MT, Bierbaum B. A new experience with alumina-on-alumina ceramic bearings for total hip arthroplasty. The Journal of Bone and Joint Surgery (Proceedings) [Internet]. 2004; 86-b(Supp_i):[4-d pp.]. Available from: http://www.bjjprocs.boneandjoint.org.uk/content/86-B/SUPP_I/4.5.short.
22. D'Antonio J, Capello W, Manley M, Naughton M, Sutton K. Alumina ceramic bearings for total hip arthroplasty: five-year results of a prospective randomized study. Clinical Orthopaedics & Related Research. 2005(436):164-71.
23. D'Antonio JA, Capello WN, Manley MT, Naughton M, Sutton K. A titanium-encased alumina ceramic bearing for total hip arthroplasty: 3- to 5-year results. Clinical orthopaedics and related research [Internet]. 2005; 441:[151-8 pp.]. Available from: http://onlinelibrary.wiley.com/o/cochrane/clcentral/articles/333/CN-00532333/frame.html http://graphics.tx.ovid.com/ovftpdfs/FPDDNCDCIHHJMB00/fs046/ovft/live/gv023/00003086/00003086-200512000-00025.pdf.
24. D'Antonio JA, Capello WN, Bierbaum B, Manley M, Naughton M. Ceramic-on-Ceramic Bearings for Total Hip Arthroplasty: 5-9 Year Follow-Up. Seminars in Arthroplasty. 2006;17(3-4):146-52.

25. D'Antonio JA, Capello WN, Naughton M. Ceramic bearings for total hip arthroplasty have high survivorship at 10 years. Clinical orthopaedics and related research [Internet]. 2012; 470(2):[373-81 pp.]. Available from: http://onlinelibrary.wiley.com/o/cochrane/clcentral/articles/719/CN-00840719/frame.html
http://www.ncbi.nlm.nih.gov/pmc/articles/PMC3254761/pdf/11999_2011_Article_2076.pdf.
26. Mesko JW, D'Antonio JA, Capello WN, Bierbaum BE, Naughton M. Ceramic-on-ceramic hip outcome at a 5- to 10-year interval: has it lived up to its expectations? Journal of arthroplasty [Internet]. 2011; 26(2):[172-7 pp.]. Available from: http://onlinelibrary.wiley.com/o/cochrane/clcentral/articles/278/CN-00779278/frame.html
http://ac.els-cdn.com/S0883540310002615/1-s2.0-S0883540310002615-main.pdf?_tid=de35b530-b2bd-11e4-a6fc-00000aab0f26&acdnat=1423749107_c4adccf9d467f4bf0d1d84558deca281.
27. Dahlstrand H, Stark A, Anissian L, Hailer NP. Elevated serum concentrations of cobalt, chromium, nickel, and manganese after metal-on-metal alloarthroplasty of the hip: a prospective randomized study. Journal of Arthroplasty. 2009;24(6):837-45.
28. Desmarchelier R, Viste A, Chouteau J, Lerat JL, Fessy MH. Metasul vs Cerasul bearings: a prospective, randomized study at 9 years. Journal of arthroplasty [Internet]. 2013; 28(2):[296-302 pp.]. Available from: http://onlinelibrary.wiley.com/o/cochrane/clcentral/articles/553/CN-00912553/frame.html
http://ac.els-cdn.com/S088354031200349X/1-s2.0-S088354031200349X-main.pdf?_tid=7cbbbd90-b2bd-11e4-ab72-00000aab0f6b&acdnat=1423748943_821f295638d07bc3e7587942ae0e5c28.
29. Digas G, Karrholm J, Thanner J, Malchau H, Herberts P. Highly cross-linked polyethylene in cemented THA: randomized study of 61 hips. Clinical Orthopaedics & Related Research. 2003(417):126-38.
30. Digas G, Karrholm J, Thanner J, Malchau H, Herberts P. The Otto Aufranc Award. Highly cross-linked polyethylene in total hip arthroplasty: randomized evaluation of penetration rate in cemented and uncemented sockets using radiostereometric analysis. Clinical Orthopaedics & Related Research. 2004(429):6-16.
31. Digas G. New polymer materials in total hip arthroplasty. Evaluation with radiostereometry, bone densitometry, radiography and clinical parameters. Acta Orthopaedica, Supplement. 2005;76(315):3-82.
32. Digas G, Karrholm J, Thanner J, Herberts P. 5-year experience of highly cross-linked polyethylene in cemented and uncemented sockets: two randomized studies using radiostereometric analysis. Acta Orthopaedica. 2007;78(6):746-54.
33. Johanson PE, Digas G, Herberts P, Thanner J, Karrholm J. Highly crosslinked polyethylene does not reduce aseptic loosening in cemented THA 10-year findings of a randomized study. Clinical Orthopaedics & Related Research. 2012;470(11):3083-93.
34. Dorr LD, Long WT, Sirianni L, Campana M, Wan Z. The argument for the use of Metasul as an articulation surface in total hip replacement. Clinical Orthopaedics & Related Research. 2004(429):80-5.
35. Eggli S, z'Brun S, Gerber C, Ganz R. Comparison of polyethylene wear with femoral heads of 22 mm and 32 mm. A prospective, randomised study. Journal of Bone & Joint Surgery - British Volume. 2002;84(3):447-51.
36. Engh CA, Jr., Stepniewski AS, Ginn SD, Beykirch SE, Sychterz-Terefenko CJ, Hopper RH, Jr., Engh CA. A randomized prospective evaluation of outcomes after total hip arthroplasty using cross-linked marathon and non-cross-linked Enduron polyethylene liners. Journal of Arthroplasty. 2006;21(6 Suppl 2):17-25.
37. Engh CA, Jr., Hopper RH, Jr., Huynh C, Ho H, Sritulanondha S, Engh CA, Sr. A prospective, randomized study of cross-linked and non-cross-linked polyethylene for total hip arthroplasty at 10-year follow-up. Journal of Arthroplasty. 2012;27(8 Suppl):2-7.e1.
38. Engh CA, MacDonald SJ, Sritulanondha S, Thompson A, Naudie D, Engh CA. 2008 John Charnley award: metal ion levels after metal-on-metal total hip arthroplasty: a randomized trial. Clinical

orthopaedics and related research [Internet]. 2009; 467(1):[101-11 pp.]. Available from: http://onlinelibrary.wiley.com/o/cochrane/clcentral/articles/041/CN-00668041/frame.html http://www.ncbi.nlm.nih.gov/pmc/articles/PMC2600985/pdf/11999_2008_Article_540.pdf.

39. Engh CA, MacDonald SJ, Sritulanondha S, Korczak A, Naudie D, Engh C. Metal ion levels after metal-on-metal total hip arthroplasty: a five-year, prospective randomized trial. Journal of Bone & Joint Surgery - American Volume. 2014;96(6):448-55.

40. Engh CA, Jr., Sritulanondha S, Korczak A, Whalen TD, Naudie DD, McCalden RW, MacDonald SJ. No Difference in Reoperations at 2 Years Between Ceramic-on-metal and Metal-on-metal THA: A Randomized Trial. Clinical Orthopaedics & Related Research. 2016;474(2):447-55.

41. Garbuz DS, Tanzer M, Greidanus NV, Masri BA, Duncan CP. The John Charnley Award: Metal-on-metal hip resurfacing versus large-diameter head metal-on-metal total hip arthroplasty: a randomized clinical trial. Clinical orthopaedics and related research [Internet]. 2010; 468(2):[318-25 pp.]. Available from: http://onlinelibrary.wiley.com/o/cochrane/clcentral/articles/677/CN-00733677/frame.html http://www.ncbi.nlm.nih.gov/pmc/articles/PMC2806981/pdf/11999_2009_Article_1029.pdf.

42. García-Rey E, García-Cimbrelo E, Cruz-Pardos A, Ortega-Chamarro J. New polyethylenes in total hip replacement: a prospective, comparative clinical study of two types of liner. Journal of bone and joint surgery British volume [Internet]. 2008; 90(2):[149-53 pp.]. Available from: http://onlinelibrary.wiley.com/o/cochrane/clcentral/articles/729/CN-00637729/frame.html http://www.bjj.boneandjoint.org.uk/content/90-B/2/149.full.pdf.

43. Garcia-Rey E, Garcia-Cimbrelo E, Cruz-Pardos A. New polyethylenes in total hip replacement: A ten- to 12-year follow-up study. Bone & Joint Journal. 2013;95-B(3):326-32.

44. Garellick G, Malchau H, Herberts P. Survival of hip replacements. A comparison of a randomized trial and a registry. Clinical Orthopaedics & Related Research. 2000(375):157-67.

45. Garellick G, Malchau H, Herberts P. The Charnley versus the Spectron hip prosthesis: clinical evaluation of a randomized, prospective study of 2 different hip implants. Journal of Arthroplasty. 1999;14(4):407-13.

46. Garellick G, Malchau H, Regner H, Herberts P. The Charnley versus the Spectron hip prosthesis: radiographic evaluation of a randomized, prospective study of 2 different hip implants. Journal of Arthroplasty. 1999;14(4):414-25.

47. Gauthier L, Dinh L, Beaule PE. Peri-acetabular bone mineral densityin total hip replacement. Bone & joint research. 2013;2(8):140-8.

48. Geerdink CH, Grimm B, Ramakrishnan R, Rondhuis J, Verburg AJ, Tonino AJ. Crosslinked polyethylene compared to conventional polyethylene in total hip replacement: pre-clinical evaluation, in-vitro testing and prospective clinical follow-up study. Acta Orthopaedica. 2006;77(5):719-25.

49. Geerdink CH, Grimm B, Vencken W, Heyligers IC, Tonino AJ. Cross-linked compared with historical polyethylene in THA: an 8-year clinical study. Clinical Orthopaedics & Related Research. 2009;467(4):979-84.

50. Girard J, Lavigne M, Vendittoli PA, Roy AG. Biomechanical reconstruction of the hip: a randomised study comparing total hip resurfacing and total hip arthroplasty. Journal of bone and joint surgery British volume [Internet]. 2006; 88(6):[721-6 pp.]. Available from: http://onlinelibrary.wiley.com/o/cochrane/clcentral/articles/553/CN-00556553/frame.html http://www.bjj.boneandjoint.org.uk/content/88-B/6/721.full.pdf.

51. Girard J, Vendittoli PA, Roy AG, Lavigne M. [Femoral offset restauration and clinical function after total hip arthroplasty and surface replacement of the hip: a randomized study]. Revue de Chirurgie Orthopedique et Reparatrice de l Appareil Moteur. 2008;94(4):376-81.

52. Lavigne M, Masse V, Girard J, Roy AG, Vendittoli PA. [Return to sport after hip resurfacing or total hip arthroplasty: a randomized study]. Revue de Chirurgie Orthopedique et Reparatrice de l Appareil Moteur. 2008;94(4):361-7.

53. Rama KRBS, Vendittoli P-A, Ganapathi M, Borgmann R, Roy A, Lavigne M. Heterotopic ossification after surface replacement arthroplasty and total hip arthroplasty: a randomized study. J Arthroplasty. 2009;24(2):256-62.
54. Vendittoli PA, Lavigne M, Girard J, Roy AG. A randomised study comparing resection of acetabular bone at resurfacing and total hip replacement. Journal of Bone & Joint Surgery - British Volume. 2006;88(8):997-1002.
55. Vendittoli PA, Lavigne M, Roy AG, Lusignan D. A prospective randomized clinical trial comparing metal-on-metal total hip arthroplasty and metal-on-metal total hip resurfacing in patients less than 65 years old. Hip Int. 2006;16(SUPPL. 4):S73-S81.
56. Vendittoli PA, Ganapathi M, Roy AG, Lusignan D, Lavigne M. A comparison of clinical results of hip resurfacing arthroplasty and 28 mm metal on metal total hip arthroplasty: a randomised trial with 3-6 years follow-up. Hip Int. 2010;20(1):1-13.
57. Vendittoli PA, Roy A, Mottard S, Girard J, Lusignan D, Lavigne M. Metal ion release from bearing wear and corrosion with 28 mm and large-diameter metal-on-metal bearing articulations: a follow-up study. Journal of Bone & Joint Surgery - British Volume. 2010;92(1):12-9.
58. Vendittoli PA, Riviere C, Roy AG, Barry J, Lusignan D, Lavigne M. Metal-on-metal hip resurfacing compared with 28-mm diameter metal-on-metal total hip replacement: a randomised study with six to nine years' follow-up. Bone & Joint Journal. 2013;95-B(11):1464-73.
59. Glyn-Jones S, Isaac S, Hauptfleisch J, McLardy-Smith P, Murray DW, Gill HS. Does highly cross-linked polyethylene wear less than conventional polyethylene in total hip arthroplasty? A double-blind, randomized, and controlled trial using roentgen stereophotogrammetric analysis. Journal of Arthroplasty. 2008;23(3):337-43.
60. Glyn-Jones S, McLardy-Smith P, Gill HS, Murray DW. The creep and wear of highly cross-linked polyethylene: a three-year randomised, controlled trial using radiostereometric analysis. Journal of Bone & Joint Surgery - British Volume. 2008;90(5):556-61.
61. Thomas GE, Simpson DJ, Mehmood S, Taylor A, McLardy-Smith P, Gill HS, Murray DW, Glyn-Jones S. The seven-year wear of highly cross-linked polyethylene in total hip arthroplasty: a double-blind, randomized controlled trial using radiostereometric analysis. Journal of bone and joint surgery American volume [Internet]. 2011; 93(8):[716-22 pp.]. Available from: http://onlinelibrary.wiley.com/o/cochrane/clcentral/articles/819/CN-00788819/frame.html http://jbjs.org/content/jbjsam/93/8/716.full.pdf.
62. Glyn-Jones S, Thomas GER, Garfjeld-Roberts P, Gundle R, Taylor A, McLardy-Smith P, Murray DW. The John Charnley Award: Highly crosslinked polyethylene in total hip arthroplasty decreases long-term wear: a double-blind randomized trial. Clinical orthopaedics and related research. 2015;473(2):432-8.
63. Grübl A, Weissinger M, Brodner W, Gleiss A, Giurea A, Gruber M, Pöll G, Meisinger V, Gottsauner-Wolf F, Kotz R. Serum aluminium and cobalt levels after ceramic-on-ceramic and metal-on-metal total hip replacement. Journal of bone and joint surgery British volume [Internet]. 2006; 88(8):[1003-5 pp.]. Available from: http://onlinelibrary.wiley.com/o/cochrane/clcentral/articles/944/CN-00566944/frame.html http://www.bjj.boneandjoint.org.uk/content/88-B/8/1003.full.pdf.
64. Haddad FS, Konan S, Tahmassebi J. A prospective comparative study of cementless total hip arthroplasty and hip resurfacing in patients under the age of 55 years: a ten-year follow-up. Bone Joint J. 2015;97-B(5):617-22.
65. Benjamin-Laing H, Tahmassebi J, Haddad F. 10 Year Follow-Up of a Prospective Randomised Study of Cementless Total Hip Replacement Versus Hip Resurfacing in Young Adults Under the Age of 55. Bone Joint J. 2013;95-B(Supp 15):13.
66. Hailer NP, Blaheta RA, Dahlstrand H, Stark A. Elevation of circulating HLA DR(+) CD8(+) T-cells and correlation with chromium and cobalt concentrations 6 years after metal-on-metal hip arthroplasty. Acta Orthopaedica. 2011;82(1):6-12.

67. Hamilton WG, McAuley JP, Dennis DA, Murphy JA, Blumenfeld TJ, Politi J. THA with Delta ceramic on ceramic: results of a multicenter investigational device exemption trial. Clinical orthopaedics and related research [Internet]. 2010; 468(2):[358-66 pp.]. Available from: http://onlinelibrary.wiley.com/o/cochrane/clcentral/articles/053/CN-00732053/frame.html http://www.ncbi.nlm.nih.gov/pmc/articles/PMC2807004/pdf/11999_2009_Article_1091.pdf.
68. Hanna SA, Sewell MD, Sri-Ram K, Miles J, Aston WJ, Pollock RC, Carrington RW, Briggs TW. The effect of femoral head size on functional outcome in primary total hip arthroplasty: a single-blinded randomised controlled trial. Hip Int. 2012;22(6):592-7.
69. Howie DW, McGee MA, Costi K, Graves SE. Metal-on-metal resurfacing versus total hip replacement-the value of a randomized clinical trial. Orthopedic Clinics of North America. 2005;36(2):195-201, ix.
70. Howie DW, Holubowycz OT, Middleton R. Large femoral heads decrease the incidence of dislocation after total hip arthroplasty: a randomized controlled trial. Journal of bone and joint surgery American volume [Internet]. 2012; 94(12):[1095-102 pp.]. Available from: http://onlinelibrary.wiley.com/o/cochrane/clcentral/articles/941/CN-00968941/frame.html http://jbjs.org/content/jbjsam/94/12/1095.full.pdf.
71. Jacobs M, Gorab R, Mattingly D, Trick L, Southworth C. Three- to six-year results with the Ultima metal-on-metal hip articulation for primary total hip arthroplasty. Journal of Arthroplasty. 2004;19(7 Suppl 2):48-53.
72. Jassim SS, Patel S, Wardle N, Tahmassebi J, Middleton R, Shardlow DL, Stephen A, Hutchinson J, Haddad FS. Five-year comparison of wear using oxidised zirconium and cobalt-chrome femoral heads in total hip arthroplasty: a multicentre randomised controlled trial. Bone Joint J. 2015;97-B(7):883-9.
73. Haddad F. Femoral head materials: no differences in outcomes - opposes. Bone Joint J. 2015;97-B(Supp 1 3).
74. Jassim SS, Patel S, Wardle N, Tahmassebi J, Middleton R, Shardlow D, Stephen A, Hutchinson J, Haddad F. Oxidized Zirconium Femoral Heads - a Multicentre Randomised Control Wear Analysis Study. Bone Joint J. 2013;95-B(Supp 15):186.
75. Jensen C, Aagaard P, Overgaard S. Recovery in mechanical muscle strength following resurfacing vs standard total hip arthroplasty - a randomised clinical trial. Osteoarthritis & Cartilage. 2011;19(9):1108-16.
76. Jensen C, Aagaard P, Overgaard S. Recovery in horizontal gait after hip resurfacing vs. total hip arthroplasty at 6-month follow-up - A randomized clinical trial. Osteoarthritis and Cartilage. 2012;20:S99.
77. Penny JO, Ovesen O, Varmarken JE, Overgaard S. Similar range of motion and function after resurfacing large-head or standard total hip arthroplasty: 2-year results from a randomized clinical trial. Acta orthopaedica [Internet]. 2013; 84(3):[246-53 pp.]. Available from: http://onlinelibrary.wiley.com/o/cochrane/clcentral/articles/200/CN-00906200/frame.html http://www.ncbi.nlm.nih.gov/pmc/articles/PMC3715815/pdf/ORT-84-246.pdf.
78. Penny JO, Varmarken JE, Ovesen O, Nielsen C, Overgaard S. Metal ion levels and lymphocyte counts: ASR hip resurfacing prosthesis vs. standard THA: 2-year results from a randomized study. Acta Orthopaedica. 2013;84(2):130-7.
79. Kadar T, Hallan G, Aamodt A, Indrekvam K, Badawy M, Skredderstuen A, Havelin LI, Stokke T, Haugan K, Espehaug B, Furnes O. Wear and migration of highly cross-linked and conventional cemented polyethylene cups with cobalt chrome or Oxinium femoral heads: a randomized radiostereometric study of 150 patients. Journal of orthopaedic research [Internet]. 2011; 29(8):[1222-9 pp.]. Available from: http://onlinelibrary.wiley.com/o/cochrane/clcentral/articles/452/CN-00801452/frame.html http://onlinelibrary.wiley.com/store/10.1002/jor.21389/asset/21389_ftp.pdf?v=1&t=i627a25f&s=eb2745b569ed6873b3c14b92ef22d4c0dfba28e2.

80. Kadar T, Hallan G, Aamodt A, Indrekvam K, Badawy M, Havelin LI, Stokke T, Haugan K, Espehaug B, Furnes O. A randomized study on migration of the Spectron EF and the Charnley flanged 40 cemented femoral components using radiostereometric analysis at 2 years. Acta orthopaedica [Internet]. 2011; 82(5):[538-44 pp.]. Available from: http://onlinelibrary.wiley.com/o/cochrane/clcentral/articles/467/CN-00804467/frame.html http://www.ncbi.nlm.nih.gov/pmc/articles/PMC3242949/pdf/ORT-1745-3674-82-538.pdf.

81. Jonsson BA, Kadar T, Havelin LI, Haugan K, Espehaug B, Indrekvam K, Furnes O, Hallan G. Oxinium modular femoral heads do not reduce polyethylene wear in cemented total hip arthroplasty at five years: a randomised trial of 120 hips using radiostereometric analysis. Bone Joint J. 2015;97-B(11):1463-9.

82. Kelley SS, Lachiewicz PF, Hickman JM, Paterno SM. Relationship of femoral head and acetabular size to the prevalence of dislocation. Clinical Orthopaedics & Related Research. 1998(355):163-70.

83. Kraay MJ, Thomas RD, Rimnac CM, Fitzgerald SJ, Goldberg VM. Zirconia versus Co-Cr femoral heads in total hip arthroplasty: early assessment of wear. Clinical orthopaedics and related research [Internet]. 2006; 453:[86-90 pp.]. Available from: http://onlinelibrary.wiley.com/o/cochrane/clcentral/articles/637/CN-00578637/frame.html http://graphics.tx.ovid.com/ovftpdfs/FPDDNCDCIHHJMB00/fs046/ovft/live/gv023/00003086/00003086-200612000-00015.pdf.

84. Langlois J, Atlan F, Scemama C, Courpied JP, Hamadouche M. A randomised controlled trial comparing highly cross-linked and contemporary annealed polyethylene after a minimal eight-year follow-up in total hip arthroplasty using cemented acetabular components. Bone & Joint Journal. 2015;97-B(11):1458-62.

85. Lavigne M, Therrien M, Nantel J, Roy A, Prince F, Vendittoli PA. The John Charnley Award: The functional outcome of hip resurfacing and large-head THA is the same: a randomized, double-blind study. Clinical orthopaedics and related research [Internet]. 2010; 468(2):[326-36 pp.]. Available from: http://onlinelibrary.wiley.com/o/cochrane/clcentral/articles/183/CN-00729183/frame.html http://www.ncbi.nlm.nih.gov/pmc/articles/PMC2807020/pdf/11999_2009_Article_938.pdf.

86. Lewis PM, Moore CA, Olsen M, Schemitsch EH, Waddell JP. Comparison of mid-term clinical outcomes after primary total hip arthroplasty with Oxinium vs cobalt chrome femoral heads. Orthopedics. 2008;31(12 Suppl 2).

87. Lindalen E, Nordsletten L, Hovik O, Rohrl SM. E-vitamin infused highly cross-linked polyethylene: RSA results from a randomised controlled trial using 32 mm and 36 mm ceramic heads. Hip Int. 2015;25(1):50-5.

88. Lombardi AV, Jr., Mallory TH, Alexiades MM, Cuckler JM, Faris PM, Jaffe KA, Keating EM, Nelson CL, Jr., Ranawat CS, Williams J, Wixson R, Hartman JF, Capps SG, Kefauver CA. Short-term results of the M2a-taper metal-on-metal articulation. Journal of Arthroplasty. 2001;16(8 Suppl 1):122-8.

89. Lombardi AV, Jr., Mallory TH, Cuckler JM, Williams J, Berend KR, Smith TM. Mid-term results of a polyethylene-free metal-on-metal articulation. Journal of Arthroplasty. 2004;19(7 Suppl 2):42-7.

90. Lombardi AV, Berend KR, Seng BE, Clarke IC, Adams JB. Delta ceramic-on-alumina ceramic articulation in primary THA: prospective, randomized FDA-IDE study and retrieval analysis. Clinical orthopaedics and related research [Internet]. 2010; 468(2):[367-74 pp.]. Available from: http://onlinelibrary.wiley.com/o/cochrane/clcentral/articles/805/CN-00730805/frame.html http://www.ncbi.nlm.nih.gov/pmc/articles/PMC2807003/pdf/11999_2009_Article_1143.pdf.

91. MacDonald SJ, McCalden RW, Chess DG, Bourne RB, Rorabeck CH, Cleland D, Leung F. Metal-on-metal versus polyethylene in hip arthroplasty: a randomized clinical trial. Clinical Orthopaedics & Related Research. 2003(406):282-96.

92. Malviya A, Ramaskandhan JR, Bowman R, Hashmi M, Holland JP, Kometa S, Lingard E. What advantage is there to be gained using large modular metal-on-metal bearings in routine primary hip

replacement? A preliminary report of a prospective randomised controlled trial. Journal of Bone & Joint Surgery - British Volume. 2011;93(12):1602-9.

93. Marston RA, Cobb AG, Bentley G. Stanmore compared with Charnley total hip replacement. A prospective study of 413 arthroplasties. Journal of Bone & Joint Surgery - British Volume. 1996;78(2):178-84.

94. Marston RA, Cobb AG, Bentley G. Does an ideal femoral head size exist for total hip arthroplasty? [abstract]. J-Bone-Joint-Surg-Br [Internet]. 1995; 77-B Suppl I:[7 p.]. Available from: http://onlinelibrary.wiley.com/o/cochrane/clcentral/articles/775/CN-00261775/frame.html.

95. Martell JM, Verner JJ, Incavo SJ. Clinical performance of a highly cross-linked polyethylene at two years in total hip arthroplasty: a randomized prospective trial. Journal of Arthroplasty. 2003;18(7 Suppl 1):55-9.

96. McCalden RW, MacDonald SJ, Rorabeck CH, Bourne RB, Chess DG, Charron KD. Wear rate of highly cross-linked polyethylene in total hip arthroplasty. A randomized controlled trial. Journal of bone and joint surgery American volume [Internet]. 2009; 91(4):[773-82 pp.]. Available from: http://onlinelibrary.wiley.com/o/cochrane/clcentral/articles/600/CN-00683600/frame.html http://jbjs.org/content/jbjsam/91/4/773.full.pdf.

97. Morison ZA, Patil S, Khan HA, Bogoch ER, Schemitsch EH, Waddell JP. A randomized controlled trial comparing Oxinium and cobalt-chrome on standard and cross-linked polyethylene. Journal of Arthroplasty. 2014;29(9 Suppl):164-8.

98. Mutimer J, Devane PA, Adams K, Horne JG. Highly crosslinked polyethylene reduces wear in total hip arthroplasty at 5 years. Clinical Orthopaedics & Related Research. 2010;468(12):3228-33.

99. Nakahara I, Nakamura N, Nishii T, Miki H, Sakai T, Sugano N. Minimum five-year follow-up wear measurement of longevity highly cross-linked polyethylene cup against cobalt-chromium or zirconia heads. Journal of arthroplasty [Internet]. 2010; 25(8):[1182-7 pp.]. Available from: http://onlinelibrary.wiley.com/o/cochrane/clcentral/articles/072/CN-00778072/frame.html http://ac.els-cdn.com/S0883540309004550/1-s2.0-S0883540309004550-main.pdf?_tid=b74d2fb6-b2bd-11e4-b333-00000aab0f6c&acdnat=1423749042_c4896983d8c4d46dc1434b4a8bcf2f54.

100. Nikolaou VS, Edwards MR, Bogoch E, Schemitsch EH, Waddell JP. A prospective randomised controlled trial comparing three alternative bearing surfaces in primary total hip replacement. Journal of Bone & Joint Surgery - British Volume. 2012;94(4):459-65.

101. Ochs U, Ilchmann T, Ochs BG, Marx J, Brunnhuber K, Luem M, Weise K. EBRA migration patterns of the Plasmacup with ceramic or polyethylene inserts: a randomised study. Zeitschrift fur Orthopadie & Unfallchirurgie. 2007;145 Suppl 1:S20-4.

102. Pabinger C, Biedermann R, Stöckl B, Fischer M, Krismer M. Migration of metal-on-metal versus ceramic-on-polyethylene hip prostheses. Clinical orthopaedics and related research [Internet]. 2003; (412):[103-10 pp.]. Available from: http://onlinelibrary.wiley.com/o/cochrane/clcentral/articles/910/CN-00438910/frame.html.

103. Penny JO, Brixen K, Varmarken JE, Ovesen O, Overgaard S. Changes in bone mineral density of the acetabulum, femoral neck and femoral shaft, after hip resurfacing and total hip replacement: two-year results from a randomised study. Journal of Bone & Joint Surgery - British Volume. 2012;94(8):1036-44.

104. Pitto RP, Schikora N, Willmann G, Graef B, Schmidt R. Radiostereoanalysis of press-fit cups with alumina liner. A randomized clinical trial Key Engineering Materials. 2003;240-242:817-22.

105. Schwammlein D, Scmidt R, Schikora N, Graef B, Willmann G, Holzwarth U, RP P. 3.1 Migration patterns of press-fit cups with polyethylene or alumina liner - a randomized clinical trial using radiostereoanalysis. Bioceramics in Joint Arthroplasty: Proceedings Proceedings 7th International Biolox Symposium. 2002:81-8.

106. Pitto RP, Blanquaert D, Hohmann D. Alternative bearing surfaces in total hip arthroplasty: zirconia-alumina pairing. Contribution or caveat? Acta Orthopaedica Belgica. 2002;68(3):242-50.

107. Poggie RA, Turgeon TR, Coutts RD. Failure analysis of a ceramic bearing acetabular component. Journal of Bone & Joint Surgery - American Volume. 2007;89(2):367-75.

108. Politi J, Dennis DA. 28 mm Ceramic-on-Ceramic Acetabular Cup Total Hip Replacement Study. ClinicalTrialsgov. 2013:NCT00208507.
109. Salemyr M, Muren O, Ahl T, Boden H, Chammout G, Stark A, Skoldenberg O. Vitamin-E diffused highly cross-linked polyethylene liner compared to standard liners in total hip arthroplasty. A randomized, controlled trial. International Orthopaedics. 2015;39(8):1499-505.
110. Schouten R, Malone AA, Tiffen C, Frampton CM, Hooper G. A prospective, randomised controlled trial comparing ceramic-on-metal and metal-on-metal bearing surfaces in total hip replacement. Journal of Bone & Joint Surgery - British Volume. 2012;94(11):1462-7.
111. Shareghi B, Johanson PE, Karrholm J. Femoral Head Penetration of Vitamin E-Infused Highly Cross-Linked Polyethylene Liners: A Randomized Radiostereometric Study of Seventy Hips Followed for Two Years. Journal of Bone & Joint Surgery - American Volume. 2015;97(16):1366-71.
112. Smolders JM, Hol A, Rijnberg WJ, van Susante JL. Metal ion levels and functional results after either resurfacing hip arthroplasty or conventional metal-on-metal hip arthroplasty. Acta Orthopaedica. 2011;82(5):559-66.
113. Smolders JM, Pakvis DF, Hendrickx BW, Verdonschot N, van Susante JL. Periacetabular bone mineral density changes after resurfacing hip arthroplasty versus conventional total hip arthroplasty. A randomized controlled DEXA study. Journal of Arthroplasty. 2013;28(7):1177-84.
114. Bisseling P, Smolders JMH, Hol A, van Susante JLC. Metal ion levels and functional results following resurfacing hip arthroplasty versus conventional small-diameter metal-on-metal total hip arthroplasty; a 3 to 5year follow-up of a randomized controlled trial. Journal of Arthroplasty. 2015;30(1):61-7.
115. Tiusanen H, Makela K, Kiilunen M, Sarantsin P, Sipola E, Pesola M. The effect of different bearing surfaces on metal ion levels in urine following 28 mm metal-on-metal and 28 mm metal-on-polyethylene total hip arthroplasty. Scandinavian Journal of Surgery: SJS. 2013;102(3):197-203.
116. Triclot P, Grosjean G, Masri F, Courpied JP, Hamadouche M. A comparison of the penetration rate of two polyethylene acetabular liners of different levels of cross-linking. A prospective randomised trial. Journal of bone and joint surgery British volume [Internet]. 2007; 89(11):[1439-45 pp.]. Available from: http://onlinelibrary.wiley.com/o/cochrane/clcentral/articles/351/CN-00628351/frame.html
http://www.bjj.boneandjoint.org.uk/content/89-B/11/1439.full.pdf.
117. Vendittoli P, Girard J, Lavigne M, Lavoie P, Duval N. Comparison of alumina-alumina to metal-polyethylene bearing surfaces in THA: a randomized study with 4- to 9-years follow-up. Acta Orthopaedica Belgica. 2007;73(4):468-77.
118. Vendittoli PA, Riviere C, Lavigne M, Lavoie P, Alghamdi A, Duval N. Alumina on alumina versus metal on conventional polyethylene: a randomized clinical trial with 9 to 15 years follow-up. Acta Orthopaedica Belgica. 2013;79(2):181-90.
119. Vendittoli PA, Duval N, Lavoie P. Prospective randomised trial comparing alumina-alumina to metal-polyethylene bearing surfaces in hybrid total hip arthroplasty. The Journal of Bone and Joint Surgery (Proceedings) [Internet]. 2005; 87-b(Supp_iii):[361-36d pp.]. Available from: http://www.bjjprocs.boneandjoint.org.uk/content/87-B/SUPP_III/361.5.short.
120. von Schewelov T, Sanzen L, Onsten I, Carlsson A, Besjakov J. Total hip replacement with a zirconium oxide ceramic femoral head: a randomised roentgen stereophotogrammetric study. Journal of Bone & Joint Surgery - British Volume. 2005;87(12):1631-5.
121. Weissinger M, Grubl A, Poll G. Serum-cobalt levels with metal-on-metal bearings in the cement-free total hip arthroplasty results covering two years; prospective study. Acta Chirurgiae Orthopaedicae et Traumatologiae Cechoslovaca. 2011;78(5):410-5.
122. Williams S, Schepers A, Isaac G, Hardaker C, Ingham E, Jagt DVD, Breckon A, Fisher J. Ceramic-on-metal hip arthroplasties: A comparative in vitro and in vivo study. Clinical Orthopaedics and Related Research. 2007(465):23-32.

123. Isaac GH, Brockett C, Breckon A, van der Jagt D, Williams S, Hardaker C, Fisher J, Schepers A. Ceramic-on-metal bearings in total hip replacement: whole blood metal ion levels and analysis of retrieved components. J Bone Joint Surg Br. 2009;91(9):1134-41.
124. Schepers A, Jagt D, Breckon A. Randomised study of four bearing surface combinations in total hip arthroplasty. Bone Joint J. 2014;96-B(Supp 13):3 4.
125. Zagra L, Anasetti F, Bianchi L, Licari V, Giacometti Ceroni R. No difference in gait recovery after THA with different head diameters: a prospective randomized study. Clinical Orthopaedics & Related Research. 2013;471(12):3830-7.
126. Zagra L, Bianchi L, Licari V, Champlon C, Giacometti Ceroni R. Gait analysis of THA with different head diameters: A prospective randomized study. Journal of Orthopaedics and Traumatology. 2011;12:S149-S50.
127. Zaoui A, Hage SE, Langlois J, Scemama C, Courpied JP, Hamadouche M. Do oxidized zirconium femoral heads reduce polyethylene wear in cemented THAs? A blinded randomized clinical trial. Clinical Orthopaedics & Related Research. 2015;473(12):3822-8.
128. Zerahn B, Borgwardt L, Ribel-Madsen S, Borgwardt A. A prospective randomised study of periprosthetic femoral bone remodeling using four different bearings in hybrid total hip arthroplasty. Hip Int [Internet]. 2011; 21(2):[176-86 pp.]. Available from: http://onlinelibrary.wiley.com/o/cochrane/clcentral/articles/825/CN-00894825/frame.html.
129. Zhou ZK, Li MG, Börlin N, Wood DJ, Nivbrant B. No increased migration in cups with ceramic-on-ceramic bearing: an RSA study. Clinical orthopaedics and related research [Internet]. 2006; 448:[39-45 pp.]. Available from: http://onlinelibrary.wiley.com/o/cochrane/clcentral/articles/385/CN-00566385/frame.html http://graphics.tx.ovid.com/ovftpdfs/FPDDNCDCIHHJMB00/fs046/ovft/live/gv023/00003086/00003086-200607000-00007.pdf.
130. Zijlstra WP, Cheung J, Sietsma MS, van Raay JJ, Deutman R. No superiority of cemented metal-on-metal vs metal-on-polyethylene THA at 5-year follow-up. Orthopedics. 2009;32(7):479.
131. Zijlstra WP, van Raay JJ, Bulstra SK, Deutman R. No superiority of cemented metal-on-metal over metal-on-polyethylene THA in a randomized controlled trial at 10-year follow-up. Orthopedics. 2010;33(3).
132. Zijlstra WP, Akker-Scheek I, Zee MJ, Raay JJ. No clinical difference between large metal-on-metal total hip arthroplasty and 28-mm-head total hip arthroplasty? International orthopaedics [Internet]. 2011; 35(12):[1771-6 pp.]. Available from: http://onlinelibrary.wiley.com/o/cochrane/clcentral/articles/476/CN-00837476/frame.html http://www.ncbi.nlm.nih.gov/pmc/articles/PMC3224629/pdf/264_2011_Article_1233.pdf.
133. Zijlstra WP, van der Veen HC, van den Akker-Scheek I, Zee MJ, Bulstra SK, van Raay JJ. Acetabular bone density and metal ions after metal-on-metal versus metal-on-polyethylene total hip arthroplasty; short-term results. Hip Int. 2014;24(2):136-43.
134. Briggs TW, Hanna SA, Kayani B, Tai S, Pollock RC, Cannon SR, Blunn GW, Carrington RW. Metal-on-polyethylene versus metal-on-metal bearing surfaces in total hip arthroplasty: a prospective randomised study investigating metal ion levels and chromosomal aberrations in peripheral lymphocytes. Bone & Joint Journal. 2015;97-B(9):1183-91.
