# Supplementary material for: Choice of implant combinations in total hip replacement: systematic review and network meta-analysis
Source: BMJ. 2017 Oct 31;359:j4651. doi: 10.1136/bmj.j4651 (PMC5683044; doi:10.1136/bmj.j4651)
Supplement: Supplementary file 5 — Appendix: Supplementary materials [file lopj039521.ww5.pdf]

Appendix 5: Risk of bias assessments in included studies

Study	**Selection bias** Random sequence generation	**Selection bias** Allocation concealment	**Performance bias** Blinding of participants	**Detection bias** Blinding of primary outcome assessors	**Attrition bias** Incomplete primary outcome data	**Reporting bias** Selective reporting	**Other sources of bias** Funding, baseline characteristics of trial arms	**Overall rating**	**Key reasons for study considered at high risk of bias**
Amanatullah et al. 2011 (1, 2)	+	+	?	+	-	?	?	-	*38% loss to follow up. Randomisation using sealed envelopes gave odd numbers: 196, 161.*
Ando et al. 2015 (3)	+	+	?	?	-	+	+	-	*Only information on patients followed up at 2 years for metal ion levels - no information on reasons patients did not attend for follow up. These could have been implant failures.*
Ayers et al. 2009 (4)	?	?	?	?	+	+	+	?	
Bal et al. 2005 (5-7)	+	+	?	?	+	+	?	?	
Bascarevic et al, 2010 (8)	+	?	?	?	+	+	?	?	
Beaupre et al. 2013 (9, 10)	+	+	?	?	?	+	+	?	
Bjorgul et al. 2013 (11)	+	+	+	?	+	+	+	+	
Brodner et al. 2003 (12, 13)	?	?	?	?	?	?	+	?	
Calvert et al. 2009 (14)	+	+	+	+	?	+	?	?	
D'Antonio et al. 2002 (15-26)	+	?	+	?	+	+	?	+	
Dahlstrand et al. 2009 (27)	+	?	?	+	+	+	+	+	
Desmarchelier et al. 2013 (28)	?	?	?	?	+	+	+	?	
Digas et al. 2003 (29-33)	?	?	?	?	+	?	+	?	

Study	**Selection bias** Random sequence generation	**Selection bias** Allocation concealment	**Performance bias** Blinding of participants	**Detection bias** Blinding of primary outcome assessors	**Attrition bias** Incomplete primary outcome data	**Reporting bias** Selective reporting	**Other sources of bias** Funding, baseline characteristics of trial arms	**Overall rating**	**Key reasons for study considered at high risk of bias**
Dorr et al. 2004 (34)	?	?	?	?	?	?	?	?	
Eggli et al. 2002 (35)	+	?	?	?	?	?	?	?	
Engh et al. 2006 (36, 37)	?	?	?	+	+	+	+	?	
Engh et al. 2009 (38, 39)	+	+	?	?	?	+	+	?	
Engh et al. 2015 (40)	+	+	+	?	+	+	+	+	
Garbuz et al. 2010 (41)	+	+	+	+	-	+	+	-	*30% lost to follow up*
Garcia-Rey et al. 2008 (42, 43)	+	?	?	?	+	+	+	+	
Garellick et al. 2000 (44-46)	+	+	?	+	+	+	+	+	
Gauthier et al. 2013 (47)	+	+	?	?	+	?	+	+	
Geerdink et al. 2006 (48)	+	?	+	?	?	?	+	?	
Geerdink et al. 2009 (49)	+	+	+	?	+	+	+	+	
Girard et al. 2006 (50-58)	+	+	?	?	+	+	+	+	
Glyn-Jones et al. 2008 (59-62)	+	+	+	+	+	+	+	+	

Study	**Selection bias** Random sequence generation	**Selection bias** Allocation concealment	**Performance bias** Blinding of participants	**Detection bias** Blinding of primary outcome assessors	**Attrition bias** Incomplete primary outcome data	**Reporting bias** Selective reporting	**Other sources of bias** Funding, baseline characteristics of trial arms	**Overall rating**	**Key reasons for study considered at high risk of bias**
Grubl et al. 2006 (63)	+	+	+	?	+	+	?	?	
Haddad et al. 2015 (64, 65)	?	+	?	+	?	+	-	-	*Only 24 out of 80 patients whose outcomes were reported were randomised.*
Hailer et al. 2011 (66)	+	?	?	+	-	+	+	-	*"39 patients had to be excluded from the analysis presented here because they had received additional metal implants, rendering the measurement of metal ion concentrations meaningless."*
Hamilton et al. 2010 (67)	+	+	?	?	+	?	+	+	
Hanna et al. 2012 (68, 134)	+	+	+	?	+	+	+	+	
Howie et al. 2005 (69)	+	?	?	?	+	+	?	?	
Howie et al. 2012 (70)	+	+	?	?	+	?	+	+	
Jacobs et al. 2004 (71)	?	?	?	?	-	+	+	-	*28% lost to follow up and uneven between groups.*
Jassim et al. 2015 (72-74)	+	+	+	?	+	?	?	+	
Jensen et al. 2011 (75-78)	+	+	?	?	+	+	+	+	
Kadar et al. 2011 (79-81)	+	+	?	?	?	+	+	+	
Kelley et al. 1998 (82)	?	?	?	?	?	+	+	?	
Kraay et al. 2006 (83)	?	?	?	?	?	+	+	?	

Study	**Selection bias** Random sequence generation	**Selection bias** Allocation concealment	**Performance bias** Blinding of participants	**Detection bias** Blinding of primary outcome assessors	**Attrition bias** Incomplete primary outcome data	**Reporting bias** Selective reporting	**Other sources of bias** Funding, baseline characteristics of trial arms	**Overall rating**	**Key reasons for study considered at high risk of bias**
Langlois et al. 2015 (84)	+	?	?	?	+	+	+	?	
Lavigne et al. 2010 (85)	+	+	?	?	?	+	+	+	
Lewis et al. 2008 (86)	+	+	?	?	?	+	+	?	
Lindalen et al. 2015 (87)	+	+	?	?	+	+	+	+	
Lombardi et al. 2001 (88, 89)	?	?	?	?	?	+	+	?	
Lombardi et al. 2010 (90)	+	?	?	?	+	+	–	–	*Authors reported RCT combined with a group of patients receiving one of the interventions in an initial prospective safety study.*
MacDonald et al. 2003 (91)	+	+	+	?	+	+	?	+	
Malviya et al. 2011 (92)	+	+	?	?	+	+	+	+	
Marston et al. 1996 (93, 94)	+	+	?	?	–	+	?	–	*40% loss to follow up. Attrition given for whole study and not per arm.*
Martell et al. 2003 (95)	?	+	?	?	–	–	+	–	*Revision not reported by randomised group.*
McCalden et al. 2009 (96)	+	+	+	?	+	?	+	+	
Morison et al. 2014 (97)	+	+	+	?	+	+	+	+	
Mutimer et al. 2010 (98)	?	+	+	+	+	?	?	+	
Nakahara et al. 2010 (99)	?	?	?	?	?	?	+	?	

Study	**Selection bias** Random sequence generation	**Selection bias** Allocation concealment	**Performance bias** Blinding of participants	**Detection bias** Blinding of primary outcome assessors	**Attrition bias** Incomplete primary outcome data	**Reporting bias** Selective reporting	**Other sources of bias** Funding, baseline characteristics of trial arms	**Overall rating**	**Key reasons for study considered at high risk of bias**
Nikolaou et al. 2012 (100)	+	+	+	?	?	?	+	+	
Ochs et al. 2007 (101)	+	?	?	?	?	?	?	?	
Pabinger et al. 2003 (102)	+	?	?	?	+	?	?	+	
Penny et al. 2013 (75-77, 103)	+	+	?	?	+	+	?	+	
Pitto et al. 2002 (106)	+	+	?	?	+	+	?	?	
Pitto et al. 2003 (104, 105)	?	?	?	?	+	+	?	?	
Poggie et al. 2007 (107)	?	?	?	?	?	-	+	-	*CoP revision rate not given at 72mths (although given for CoC group). Primary aim of paper was to look at risk factors for CoC failure - CoP control group does not appear to have been of particular interest and therefore not reported as such.*
Politi et al. 2013 (108)	?	?	?	?	?	?	?	?	
Salemyr et al. 2015 (109)	+	+	+	+	+	+	+	+	
Schouten et al. 2012 (110)	+	+	+	?	+	+	?	+	
Shareghi et al. 2015 (111)	?	+	+	+	+	+	+	+	

Study	**Selection bias** Random sequence generation	**Selection bias** Allocation concealment	**Performance bias** Blinding of participants	**Detection bias** Blinding of primary outcome assessors	**Attrition bias** Incomplete primary outcome data	**Reporting bias** Selective reporting	**Other sources of bias** Funding, baseline characteristics of trial arms	**Overall rating**	**Key reasons for study considered at high risk of bias**
Smolders et al. 2011 (112-114)	+	+	?	?	+	+	?	?	
Tiusanen et al. 2013 (115)	?	?	?	?	?	+	+	?	
Triclot et al. 2007 (116)	+	+	?	?	?	+	+	?	
Venditolli et al. 2007 (117-119)	+	+	+	?	+	+	?	+	
von Schewelov et al. 2005 (120)	?	?	?	?	?	?	?	?	
Weissinger et al. 2011 (121)	?	?	?	?	?	?	+	?	
Williams et al. 2007 (122-124)	?	?	?	?	?	?	?	?	
Zagra et al. 2013 (125, 126)	+	?	?	?	?	+	+	?	
Zaoui et al. 2015 (127)	?	+	?	?	+	+	?	?	
Zerahn et al. 2011 (128)	+	-	?	?	+	+	+	-	*"The patient was excluded from the study if the surgeon found another prosthesis more appropriate during surgery, according to departmental protocols, in which case a new envelope with the same bearing combination as drawn was re-entered into the pool of envelopes for subsequent use."*
Zhou et al. 2006 (129)	?	?	?	?	+	+	+	?	

Study	**Selection bias** Random sequence generation	**Selection bias** Allocation concealment	**Performance bias** Blinding of participants	**Detection bias** Blinding of primary outcome assessors	**Attrition bias** Incomplete primary outcome data	**Reporting bias** Selective reporting	**Other sources of bias** Funding, baseline characteristics of trial arms	**Overall rating**	**Key reasons for study considered at high risk of bias**
Zijlstra et al. 2009 (130, 131)	+	+	?	?	-	+	?	-	*Marked attrition at 10yr follow up, 46% follow up for MoM, 56% for MoP.*
Zijlstra et al. 2011 (132, 133)	+	+	?	?	+	+	?	?	
