# Supplementary material for: Choice of implant combinations in total hip replacement: systematic review and network meta-analysis
Source: BMJ. 2017 Oct 31;359:j4651. doi: 10.1136/bmj.j4651 (PMC5683044; doi:10.1136/bmj.j4651)
Supplement: Supplementary file 6 — Appendix: Supplementary materials [file lopj039521.ww6.pdf]

Appendix 6: Additional results for the primary outcome

Supplementary Figure 1. Pair-wise analyses: Revision at 0-2 years after primary THR

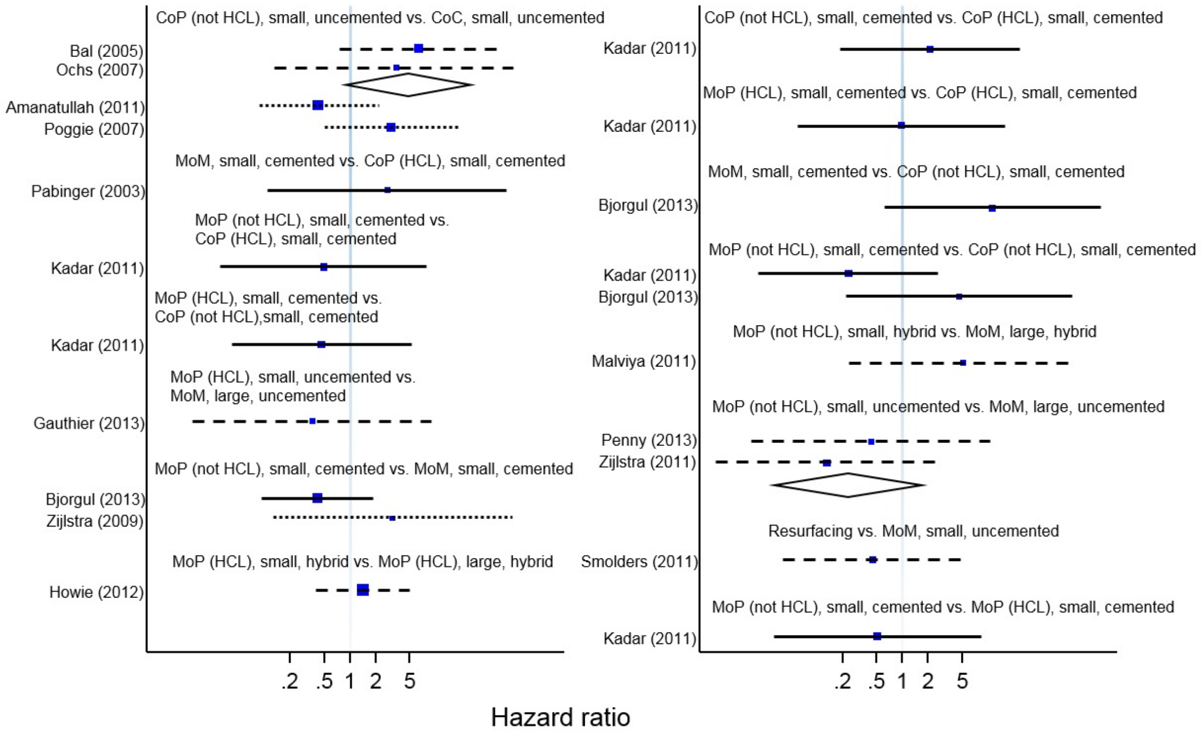

Values below 1 favour the first intervention; Solid line: included in the NMA; dotted line: High RoB; dashed line: disconnected; dotted/dashed line: zero events; MoP: metal-on-polyethylene; CoP: ceramic-on-polyethylene; CoC: ceramic-on-ceramic; MoM: metal-on-metal; HCL: highly cross-linked. Diamonds display the overall effect estimate (and its 95% CI), based on a fixed-effect model, where more than one effect estimate was available for the same implant comparison (estimates assessed at high risk of bias were excluded from these summaries).

Supplementary Figure 2. Pair-wise analyses: Revisions at 2-10 years after primary THR

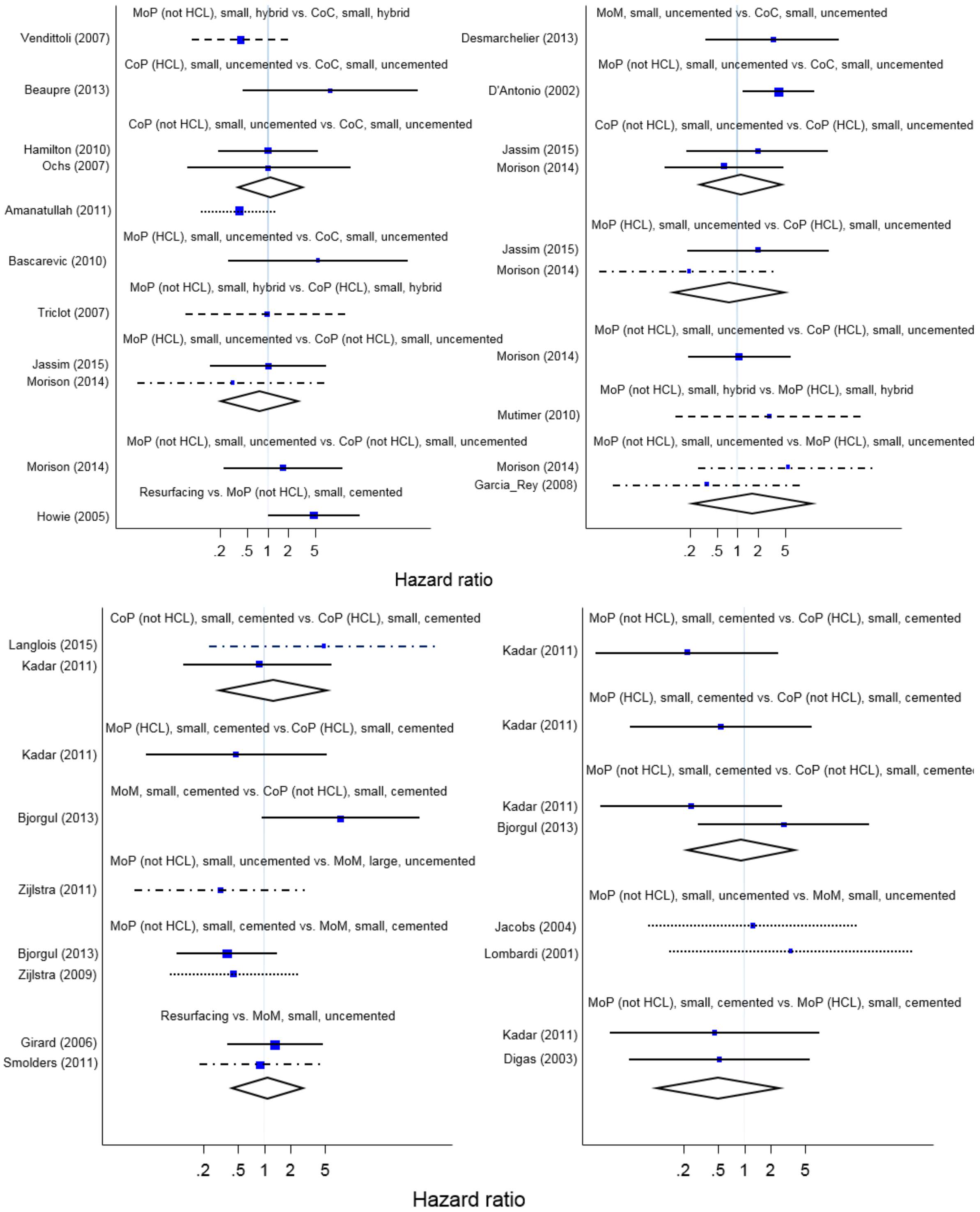

Values below 1 favour the first intervention; Solid line: included in the NMA; dotted line: High RoB; dashed line: disconnected; dotted/dashed line: zero events; MoP: metal-on-polyethylene; CoP: ceramic-on-polyethylene; CoC: ceramic-on-ceramic; MoM: metal-on-metal; HCL: highly cross-linked. Diamonds display the overall effect estimate (and its 95% CI), based on a fixed-effect model, where more than one effect estimate was available for the same implant comparison (estimates assessed at high risk of bias were excluded from these summaries).

Supplementary Figure 3. Fixed-effect NMA for revisions at each time period (same subset of studies as in the random walk model, cut-off value of 2 years)

A. 0-2 years

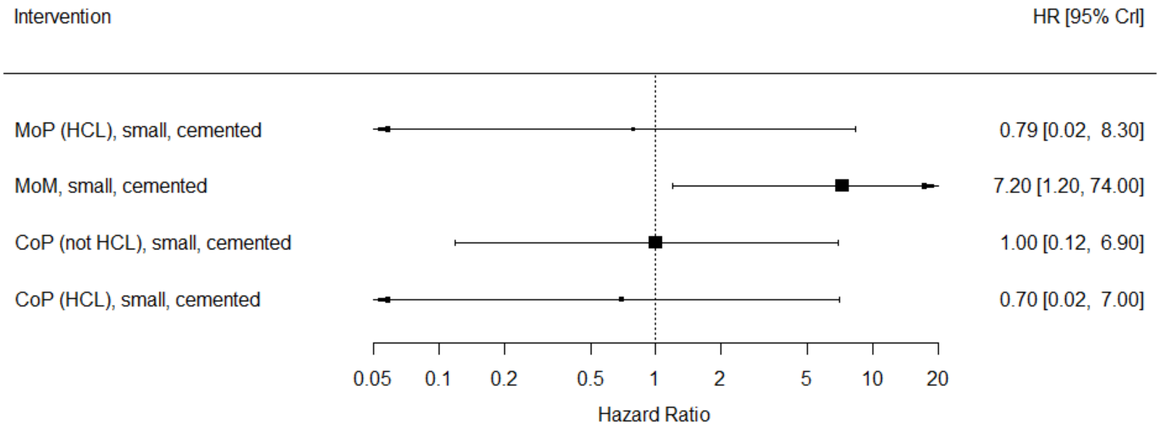

B. 2-10 years

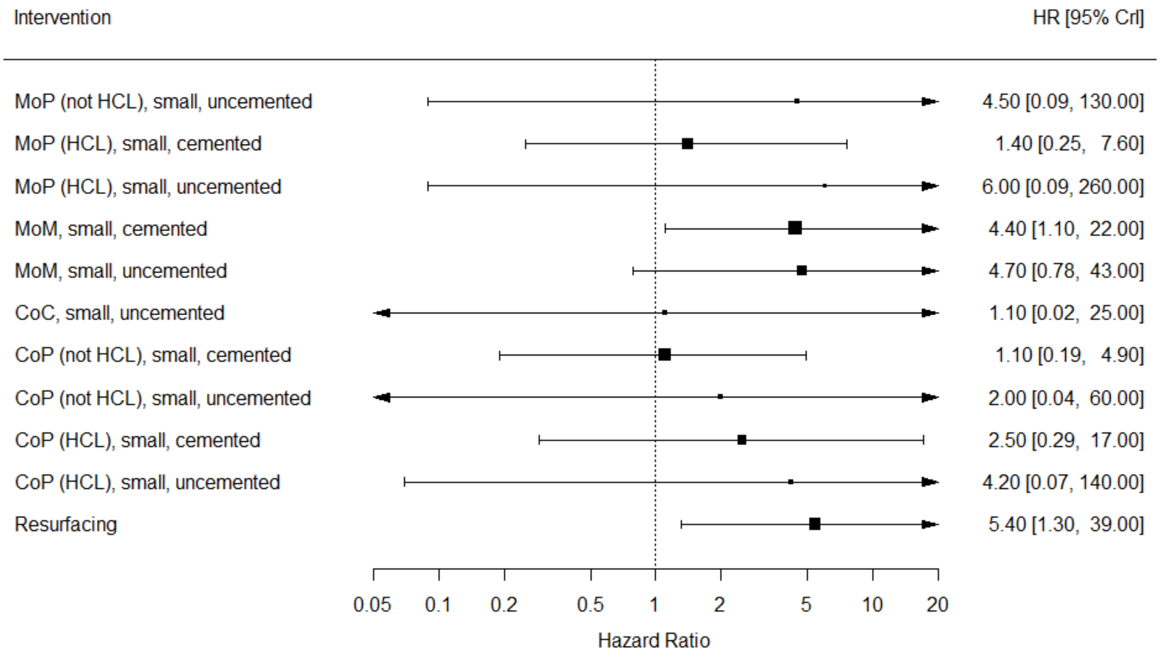

Reference: metal-on-polyethylene (not HCL), small, cemented; values above 1 favour the reference; MoP: metal-on-polyethylene; CoP: ceramic-on-polyethylene; CoC: ceramic-on-ceramic; MoM: metal-on-metal; HCL: highly cross-linked; CrI: credible interval.

Supplementary Figure 4. Network plots for revisions at 0-5 years and 5-10 years

A. 0-5 years

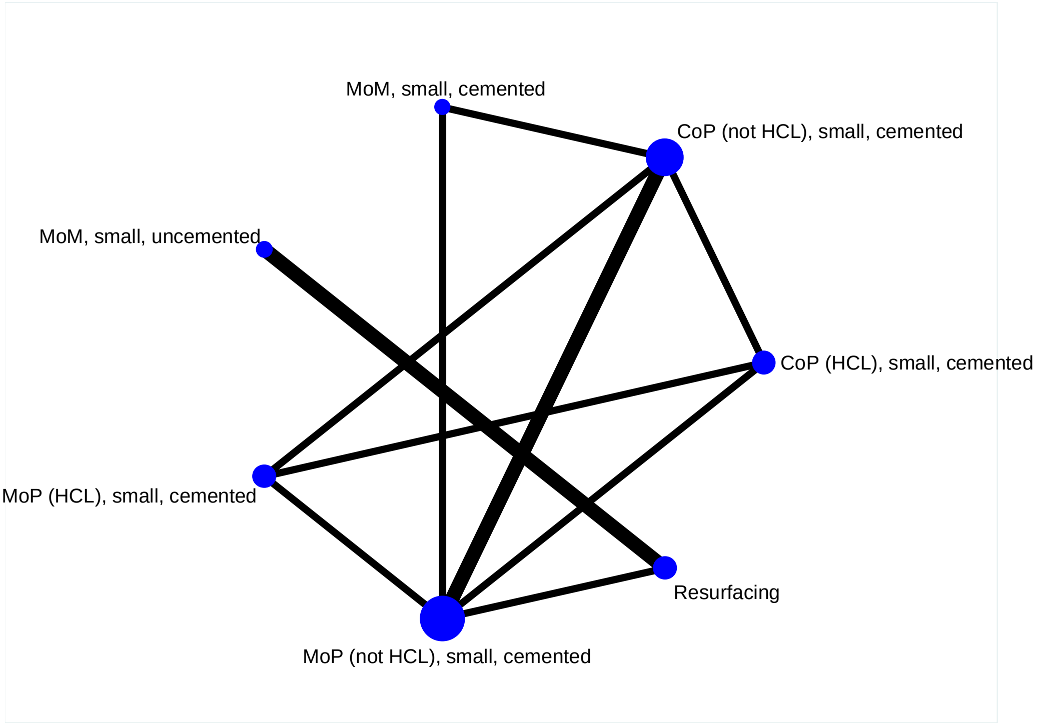

B. 5-10 years

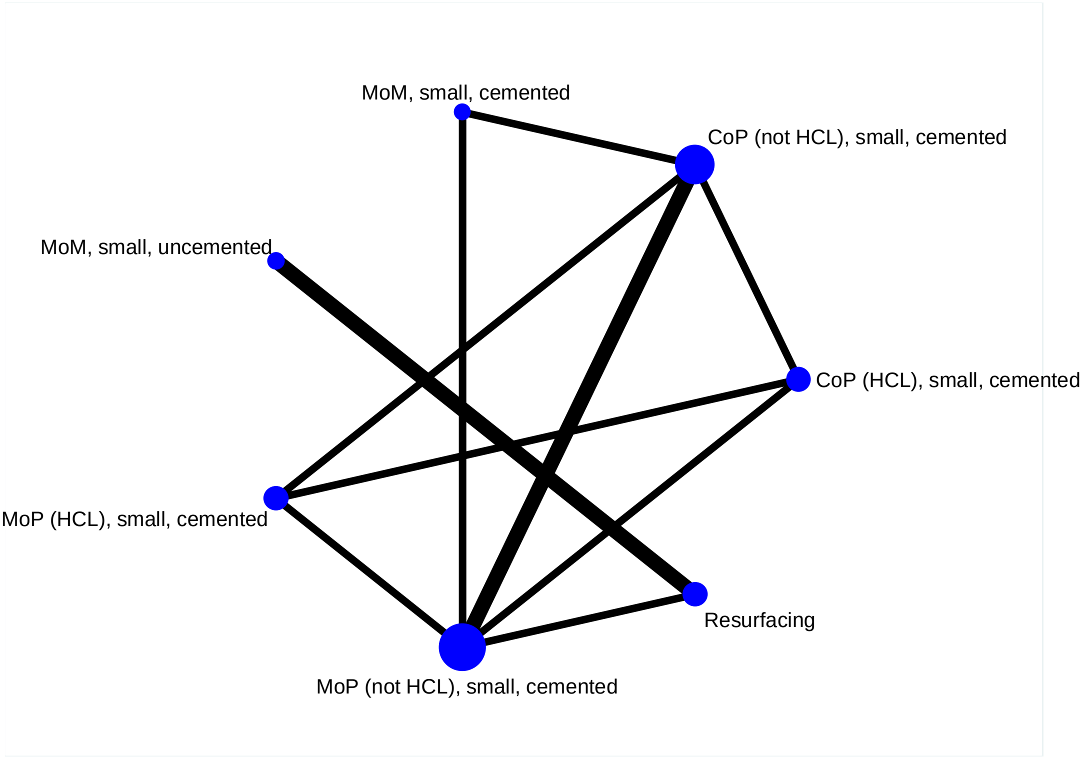

MoP: metal-on-polyethylene; CoP: ceramic-on-polyethylene; CoC: ceramic-on-ceramic; MoM: metal-on-metal; HCL: highly cross-linked. Node size and edge thickness are proportional to the number of studies contributing to each intervention and comparison, respectively.

Supplementary Figure 5. Fixed-effect NMA for revisions at each time period (same subset of studies as in the random walk model, cut-off value of 5 years)

A. 0-5 years

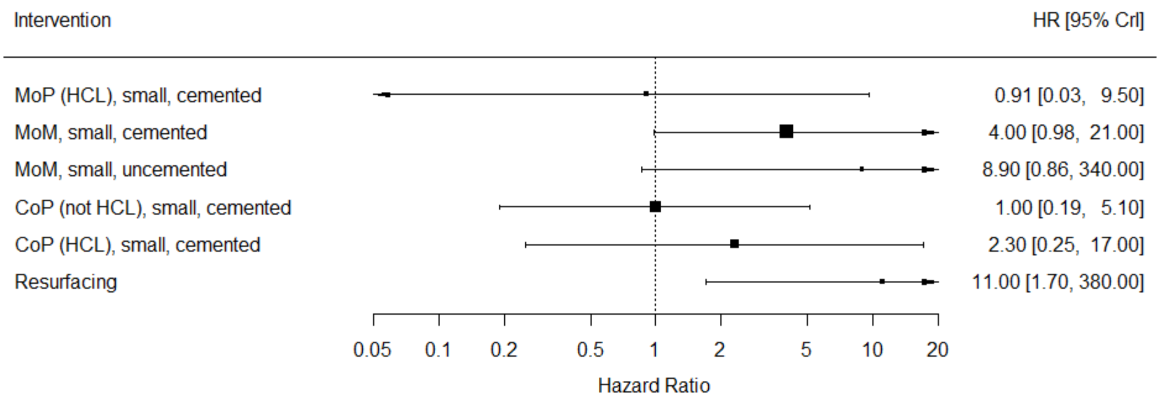

B. 5-10 years

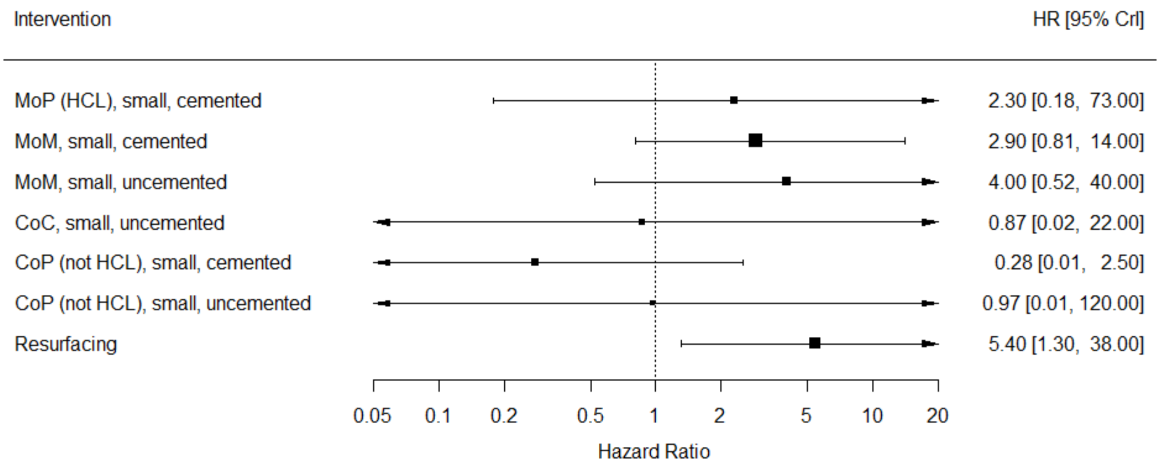

Reference: metal-on-polyethylene (not HCL), small, cemented; values above 1 favour the reference; MoP: metal-on-polyethylene; CoP: ceramic-on-polyethylene; CoC: ceramic-on-ceramic; MoM: metal-on-metal; HCL: highly cross-linked; CrI: credible interval

Supplementary Figure 6. Random walk NMA model for revisions (time periods: 0-5 & 5-10 years)

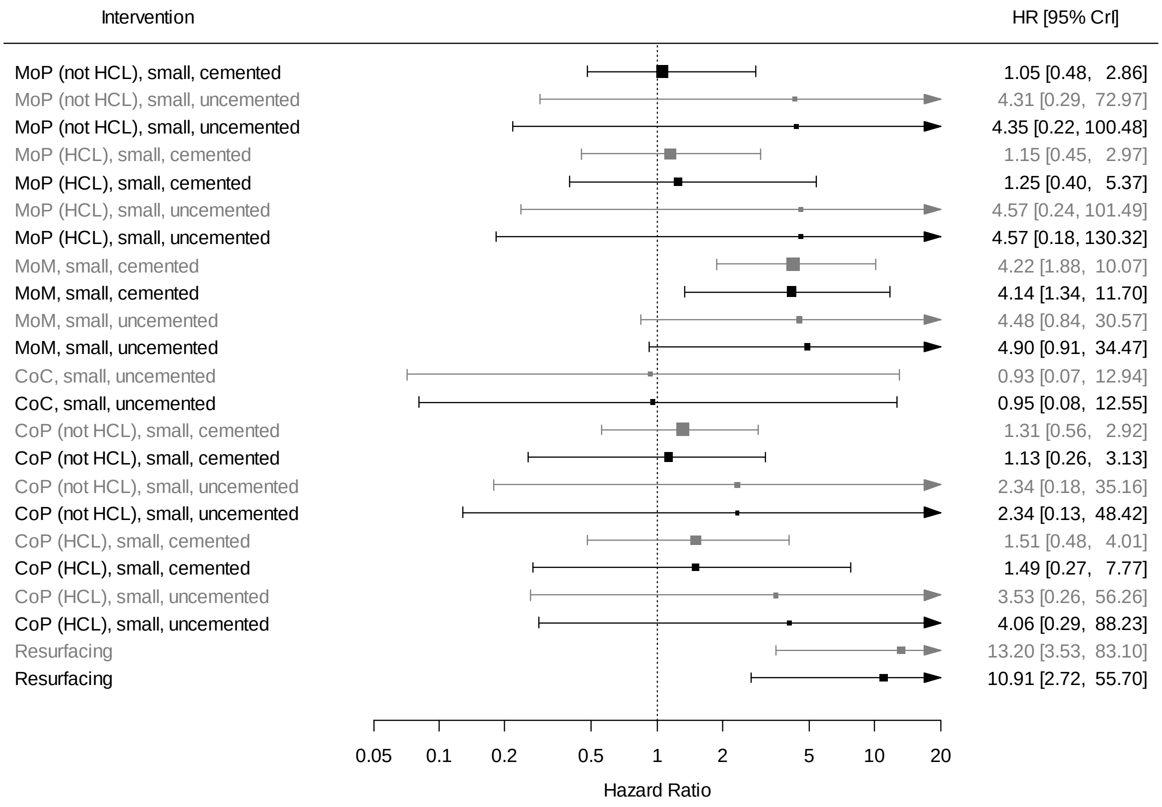

Grey: 0-5 years; Black: 5-10 years; Reference: Metal-on-polyethylene (not HCL), small, cemented, 0-5 years; Hazard ratios greater than 1 favour reference. MoP: metal-on-polyethylene; CoP: ceramic-on-polyethylene; CoC: ceramic-on-ceramic; MoM: metal-on-metal; HCL: highly cross-linked; CrI: credible interval
