# Supplementary material for: Choice of implant combinations in total hip replacement: systematic review and network meta-analysis
Source: BMJ. 2017 Oct 31;359:j4651. doi: 10.1136/bmj.j4651 (PMC5683044; doi:10.1136/bmj.j4651)
Supplement: Supplementary file 7 — Appendix: Supplementary materials [file lopj039521.ww7.pdf]

Appendix 7: WOMAC and OHS outcomes (studies at high risk of bias excluded)

**Study**<br>**Key paper (all study references)**	**Group**<br>**Bearing combination**<br>**Head size**<br>**Fixation**	**Outcome measures relevant to review**<br>**Follow up times (months)**
Ayers et al. 2009 (4)	1. MoP<br>Titanium: UHMWPE<br>28mm<br>Uncemented<br>2. MoP<br>Titanium: HXLPE<br>28mm<br>Uncemented	No significant differences in WOMAC between randomized groups at 6 months, 1 and 2 years (no p values reported)
Beaupre et al. 2013 (9, 10)	1. CoC<br>Alumina: Alumina<br>32mm<br>Uncemented<br>2. CoP<br>Alumina: HXLPE<br>28mm<br>Uncemented	No differences at 5 years between groups for WOMAC pain (p=0.74), function (p=0.97) or stiffness (p=0.44)
Engh et al. 2009 (38, 39)	1. MoP<br>Co-Cr: HXLPE<br>28mm<br>Uncemented<br>2. MoM<br>Co-Cr: Co-Cr<br>28mm<br>Uncemented<br>3: MoM<br>Co-Cr: Co-Cr<br>36mm<br>Uncemented	At 2 years, small head MoM had marginally poorer WOMAC score than MoP group (p=0.052)
Gauthier et al. 2013 (47)	1. MoM<br>Co-Cr-Mo: Co-Cr-Mo<br>36-54<br>Uncemented<br>2. MoP<br>Co-Cr: HXLPE<br>28-32mm<br>Uncemented	No difference in WOMAC domains between groups at 2 years (no p value reported)
Girard et al. 2006 (50-58)	1. Resurfacing<br>Co-Cr-Mo: Co-Cr-Mo<br>40mm<br>Hybrid<br>2. MoM<br>Co-Cr-Mo: Co-Cr-Mo<br>28mm<br>Uncemented	No difference between groups for WOMAC score at mean 27 months (p=0.409)
Glyn-Jones et al. 2008 (59-62)	1. MoP<br>Co-Cr: UHMWPE<br>28mm<br>Hybrid<br>2. MoP<br>Co-Cr: HXLPE<br>28mm<br>Hybrid	No significant difference in OHS between the groups at 10 years (p=0.42)
Hanna et al. 2012 (68)	1. MoP<br>Co-Cr: UHMWPE<br>28-32mm	No difference in OHS between groups at 12 or 36 months (p>0.05)

	Cemented	
	2. MoM<br>Co-Cr: Co-Cr<br>44-54mm<br>Hybrid	
Jassim et al. 2015 (72-74)	1. MoP<br>Co-Cr: HXLPE<br>32mm<br>Uncemented	No difference between groups in WOMAC score at 5 years (p=0.847)
	2. CoP<br>Zirconia: HXLPE<br>32mm<br>Uncemented	
	3. CoP<br>Zirconia: UHMWPE<br>32mm<br>Uncemented	
Jensen et al. 2011* (75-78)	1. Resurfacing<br>Co-Cr-Mo: Co-Cr-Mo<br>47-57mm<br>Hybrid	No significant difference between groups in WOMAC at 2 years (mean difference 1, 95%CI -8, 10)
	2. CoP<br>Alumina: Unspecified PE<br>28mm<br>Uncemented	
Lavigne et al. 2010* (85)	1. Resurfacing<br>Unspecified metal: Unspecified metal<br>40mm<br>Hybrid	WOMAC scores similar between groups at 1 year (p>0.05)
	2. MoM<br>Unspecified metal: Unspecified metal<br>40mm<br>Uncemented	
Lewis et al. 2008 (86)	1. CoP<br>Zirconium: Standard PE or HXLPE<br>Not stated<br>Uncemented	No difference between groups in WOMAC score at 1 or 2 years (p>0.159)
	2. MoP<br>Co-Cr: Standard PE or HXLPE<br>Not stated<br>Uncemented	
Lindalen et al. 2015 (87)	1. CoP<br>Zirconia/ alumina: HXLPE (vitamin E)<br>32mm<br>Uncemented	OHS similar in groups at 2 years (p=0.72)
	2. CoP<br>Zirconia/ alumina: HXLPE (vitamin E)<br>36mm<br>Uncemented	
MacDonald et al. 2003* (91)	1. MoM<br>Co-Cr-Mo: Co-Cr-Mo<br>28mm<br>Uncemented	No significant difference in WOMAC score between groups (p>0.05)
	2. MoP<br>Co-Cr-Mo: UHMWPE<br>28mm	

	Uncemented	
Malviya et al.<br>2011 (92)	1. MoM<br>Co-Cr: Unspecified metal<br>38-54mm<br>Hybrid	No significant difference between groups for WOMAC pain (p=0.296), function (p=0.303) and stiffness (p=0.093) at 2 years
	2. MoP<br>Unspecified metal:<br>Unspecified PE<br>28mm<br>Hybrid	
McCalden et al.<br>2009* (96)	1. MoP<br>Co-Cr: HXLPE<br>28mm<br>Hybrid	At mean 6.8 years no differences between groups for WOMAC score (no p value reported)
	2. MoP<br>Co-Cr: Conventional PE<br>28mm<br>Hybrid	
Morison et al.<br>2014 (97)	1. MoP<br>Co-Cr: UHMWPE<br>28mm<br>Uncemented	No differences between groups for WOMAC scores at 5 years (p>0423)
	2. MoP<br>Co-Cr: HXLPE<br>28mm<br>Uncemented	
	3. CoP<br>Zirconium: UHMWPE<br>28mm<br>Uncemented	
	4. CoP<br>Zirconium: HXLPE<br>28mm<br>Uncemented	
Nikolaou et al.<br>2012 (100)	1. MoP<br>Co-Cr: UHMWPE<br>28mm<br>Uncemented	No difference at 5 years in WOMAC pain (p=0.543), function (p=0.102) and stiffness (p=0.99)
	2. MoP<br>Co-Cr: HXLPE<br>28mm<br>Uncemented	
	3. CoC<br>Unspecified ceramic:<br>Unspecified ceramic<br>28mm<br>Uncemented	
Penny et al.<br>2013* (75-77, 103)	1. MoP<br>Co-Cr: UHMWPE or HXLPE<br>28mm<br>Uncemented	No difference in WOMAC between groups at 2 years (mean difference -5, 95% CI -11, 2)
	2. MoM<br>Co-Cr: Co-Cr<br>47-56mm<br>Uncemented	
Schouten et al.<br>2012 (110)	1. CoM<br>Zirconia/ alumina: Co-Cr-Mo<br>28-36mm<br>Uncemented	Comparable improvement in WOMAC (p=0.64) and OHS (p=0.57) between groups at 12 months
	2. MoM<br>Co-Cr-Mo: Co-Cr-Mo	

	28-36mm<br>Uncemented	
Smolders et al. 2011 (112-114)	1. Resurfacing<br>Co-Cr: Co-Cr<br>49mm<br>Hybrid	Better OHS at 24 months in resurfacing group (p=0.04)
	2. MoM<br>Co-Cr: Co-Cr<br>28mm<br>Uncemented	
Tiusanen et al. 2013 (115)	1. MoP<br>Co-Cr-Mo: Unspecified PE<br>30mm<br>Uncemented	No statistical analysis but OHS similar between groups
	2. MoM<br>Co-Cr-Mo: Co-Cr-Mo<br>30mm<br>Uncemented	
Venditolli et al. 2007 (117-119)	1. CoC<br>Alumina: Alumina<br>32mm<br>Hybrid	No significant difference in WOMAC scores between groups at mean 79 months (p=0.435)
	2. MoP<br>Stainless steel: UHMWPE<br>28mm<br>Hybrid	
Zhou et al. 2006 (129)	1. CoC<br>Alumina: Alumina<br>28mm<br>Hybrid	No difference in WOMAC between groups at 2 years (no p value reported)
	2. MoP<br>Co-Cr: HXLPE<br>28mm<br>Hybrid	
Zijlstra et al. 2011 (132, 133)	1. MoM<br>Co-Cr: Co-Cr<br>42-56<br>Uncemented	No difference between groups in OHS at 1 year (p=0.752)
	2. MoP<br>Co-Cr: UHMWPE<br>28mm<br>Uncemented	
